# Protein Profiling in Human Papillomavirus-Associated Cervical Carcinogenesis: Cornulin as a Biomarker for Disease Progression

**DOI:** 10.3390/cimb45040235

**Published:** 2023-04-20

**Authors:** Gaayathri Kumarasamy, Mohd Nazri Ismail, Sharifah Emilia Tuan Sharif, Christopher Desire, Parul Mittal, Peter Hoffmann, Gurjeet Kaur

**Affiliations:** 1 Institute for Research in Molecular Medicine (INFORMM), Universiti Sains Malaysia, Minden 11800, Pulau Pinang, Malaysia; 2 Analytical Biochemistry Research Centre (ABrC), Universiti Sains Malaysia, Bayan Lepas 11900, Pulau Pinang, Malaysia; 3 Department of Pathology, School of Medical Sciences, Universiti Sains Malaysia, Kubang Kerian 16150, Kelantan, Malaysia; 4 Clinical Health Sciences, University of South Australia, City West Campus, Adelaide, SA 5000, Australia

**Keywords:** human papillomavirus, oncogenesis, cervical cancer, proteomics, biomarker

## Abstract

Nearly 90% of cervical cancers are linked to human papillomavirus (HPV). Uncovering the protein signatures in each histological phase of cervical oncogenesis provides a path to biomarker discovery. The proteomes extracted from formalin-fixed paraffin-embedded tissues of the normal cervix, HPV16/18-associated squamous intraepithelial lesion (SIL), and squamous cell carcinoma (SCC) were compared using liquid chromatography-mass spectrometry (LC-MS). A total of 3597 proteins were identified, with 589, 550, and 1570 proteins unique to the normal cervix, SIL, and SCC groups, respectively, while 332 proteins overlapped between the three groups. In the transition from normal cervix to SIL, all 39 differentially expressed proteins were downregulated, while all 51 proteins discovered were upregulated in SIL to SCC. The binding process was the top molecular function, while chromatin silencing in the SIL vs. normal group, and nucleosome assembly in SCC vs. SIL groups was the top biological process. The PI3 kinase pathway appears crucial in initiating neoplastic transformation, while viral carcinogenesis and necroptosis are important for cell proliferation, migration, and metastasis in cervical cancer development. Annexin A2 and cornulin were selected for validation based on LC-MS results. The former was downregulated in the SIL vs. normal cervix and upregulated in the progression from SIL to SCC. In contrast, cornulin exhibited the highest expression in the normal cervix and lowest in SCC. Although other proteins, such as histones, collagen, and vimentin, were differentially expressed, their ubiquitous expression in most cells precluded further analysis. Immunohistochemical analysis of tissue microarrays found no significant difference in Annexin A2 expression between the groups. Conversely, cornulin exhibited the strongest expression in the normal cervix and lowest in SCC, supporting its role as a tumor suppressor and potential biomarker for disease progression.

## 1. Introduction

Cervical cancer is one of the most common gynecological cancers in the world. It is ranked the fourth most common cancer among women, after breast, colorectal, and lung cancer. In 2018, it caused an estimated 570,000 cases and was responsible for approximately 311,000 deaths [1]. Infection with human papillomavirus (HPV) is the most important etiological factor for cervical cancer [2], with high-risk HPV types 16 and 18 being most prevalent; they are detected in approximately 70% of cervical cancers [3,4]. Most infections are asymptomatic and spontaneously cleared by the immune system within six months to two years. However, persistent high-risk HPV infections may cause abnormal changes in epithelial cells of the cervical transformation zone, initiating the development of low- and high-grade squamous intraepithelial lesions (SIL) that may eventually progress to invasive cervical cancer [5,6]. SIL can be graded into low-grade squamous intraepithelial lesions (LSIL), and high-grade intraepithelial lesions (HSIL) [7]. LSIL usually represents the infection stage rather than the disease development stage; therefore, it does not always signify disease progression. However, HSIL can potentially progress and develop invasive cervical cancer [7]. The expression of the E6 and E7 viral oncogene proteins can interfere with the function of p53 and pRb [8,9], impacting several cellular pathways, such as apoptosis, proliferation, growth, and motility, and causing progressive genetic instability [10,11,12,13]. Therefore, it is important to understand the biological and cellular processes that can facilitate the growth of normal cells into abnormal ones.

Over the past decade, proteomics approaches have identified protein signatures that can be used for early disease diagnosis, prognosis, and/or disease progression [14]. There are several serum protein biomarkers, such as squamous cell carcinoma antigen (SCC-Ag), CYFRA 21-1 (cytokeratin 19 fragment), and carcinoembryonic antigen (CEA), that are of diagnostic and prognostic value in cervical cancer [15]. The study of the proteome comprises protein structure, expression, functions, interactions, and modifications [16]. The proteome can be completely characterized by using sensitive mass spectrometry-based techniques such as nano-liquid chromatography (nano-LC) coupled to tandem mass spectrometry.

Human tissue specimens are the most important material for translational clinical research [17], derived from fresh-frozen or formalin-fixed paraffin-embedded (FFPE) tissues [18,19]. Although fresh tissue samples are easier to process after excision, FFPE is more economical as tissues can be stored at room temperature for years while maintaining the necessary integrity for pathology analysis [20]. FFPE tissues have been successfully used for bottom-up mass spectrometry shotgun proteomics, indicating a significant advance in clinical proteomics [21,22].

Most proteomics studies found in the literature are conducted on cervical cancer, and only a few reports have examined protein markers of precancerous cervical lesions [23]. Some of the biomarkers found in precancerous lesions of cervical cancer include coagulation factor IX, complement factor I, afamin, and alpha-1-acid glycoprotein 2 [24]. However, these proteins are identified in plasma samples rather than FFPE tissues. This study aimed to identify and compare the protein profiles in FFPE-derived tissues of the normal cervix, HPV 16/18-associated precancerous SIL lesions, and SCC tissues utilizing LC-ESI-MS/MS. Here, we investigated the molecular functions, biological processes, pathways, and protein–protein interactions of the identified proteins.

Two proteins, annexin A2 and cornulin were selected based on the LC-ESI-MS/MS results, for histochemical protein expression in tissues to evaluate their role as potential biomarkers. Annexin A2 is a multifunctional calcium- and lipid-binding protein found in almost all human tissues and cells [25]. It has been linked to a variety of intracellular processes such as membrane domain organization, membrane fusion, vesicle aggregation, epithelial cell polarity, cytoskeletal–membrane dynamics, phagocytosis, exocytosis, endocytosis, and transcriptional regulation via RNA binding [26,27,28,29]. ANXA2 has been linked to cancers including ovarian, colorectal, breast and lung cancer; it has also been identified as a key mediator of HPV entry and intracellular trafficking [25]. Cornulin, encoded by the CRNN gene on chromosome 1q21, is a 495 amino acid protein that belongs to the S100 fused-type protein family and is reported to have tumor-suppressing properties [30]. Cornulin is a squamous cell-specific protein and it is found to be expressed in squamous cells of the esophagus, cervical epithelium, and skin [31]. Downregulation of cornulin has been reported to be a prominent hallmark in esophageal and head and neck carcinomas. Although annexin A2 and cornulin have been reported in other studies, their expression in cervical cancer FFPE tissues has not been reported.

## 2. Materials and Methods

### 2.1. Tissue Samples

Six cases each of normal cervix and squamous cell carcinoma (SCC) and five cases of high-grade squamous intraepithelial lesion III (SIL), were retrieved from the Department of Pathology, School of Medical Sciences, Universiti Sains Malaysia (USM) Kubang Kerian, Kelantan, Malaysia. Ethics approval and the exemption for patient’s consent were obtained from the Human Research Ethics Committee USM. (Letter dated 5 September 2019). The sample selection criteria included (i) histologically confirmed cases of the normal cervix, SIL, and SCC; (ii) FFPE tissue samples of an adequate squamous epithelial component in biopsy material from SIL and SCC lesions and normal ectocervical tissues from hysterectomy specimens that were removed for non-cervical conditions. Through immunohistochemistry using the EnVision™ FLEX kit (Dako, USA, K8023) with anti-HPV16 E6 + HPV18 E6 [C1P5] (1:200 dilution, Abcam, UK, ab70) as the primary antibody on FFPE tissue sections, SIL and SCC cases were confirmed positive for HPV types 16 or 18, and the normal cervix was confirmed HPV-negative. The selected FFPE tissue blocks were cut at 10μm-thickness, and six consecutive sections were placed on polyethylene naphthalate (PEN) membrane glass slides (Carl Zeiss, Oberkochen, Baden-Württemberg). The squamous epithelium component was excised from the tissue sections using ZEISS PALM Microbeam Laser Microdissection System (Carl Zeiss, Oberkochen, Baden-Württemberg) and transferred into separate adhesive cap vials. In total, there were 17 samples comprising 6 normal cervices, 5 HPV-associated SIL, and 6 HPV-associated SCC cases. Eight samples, representing three normal cervices, two SIL, and three SCC tissues, were sent to the University of South Australia in dry tissue form.

### 2.2. Peptide Extraction from FFPE Tissue and Mass Spectrometry Analysis

This study used two types of peptide extraction methods from FFPE tissues and two mass spectrometry analytical instruments. Briefly, the first method utilized an urea lysis buffer to extract peptides, followed by separation using Easy-nLC II nano-LC coupled to nano LC-LTQ-Orbitrap-MS/MS, located at the Analytical Biochemistry Research Centre (ABrC), Universiti Sains Malaysia. The second workflow employed *Rapi*Gest SF surfactant (Waters Corporation, Milford, MA, USA) for peptide extraction, and afterward, the sample was subjected to timsTOF mass spectrometry using DDA-PASEF (LC-TIMS-TOF MS/MS), located at Clinical Health Sciences, University of South Australia.

Using the first method, peptides were extracted from three samples in each histological group (total *n* = 9). The vials containing microdissected tissue fragments were covered with 200 µL of 10 mM citric acid (pH 6.0), incubated for 45 min at 98 °C, and then cooled at room temperature (RT) and washed with 50 µL of 25 mM ammonium bicarbonate. Then, 200 µL of 8 M urea in 0.1 M Tris/HCl, pH 8.0 (UA) was added and vortexed for 3 min. The samples were incubated with 2 µL 1 M dithiothreitol (DTT) for 30 min at RT, followed by 50 µL 50 mM iodoacetamide (IAA) for 30 min in the dark. After the samples were centrifuged for 5 min at 14,000× *g*, the buffers were discarded before washing twice with 100 µL of UA, followed by 100 µL of 0.05 M Tris/HCl pH 8.0 and 25 mM ammonium, respectively. The solution was centrifuged at 14,000× g, and the supernatant was discarded. Ammonium bicarbonate 25 mM and 10 μL 20 ng/μL modified porcine trypsin (Promega, Madison, WI, USA) were added and incubated overnight at 37 °C. To stop the reaction, 1 µL of 10% TFA was added. Subsequently, the samples were centrifuged at 14,000× *g* for 10 min, and the supernatant was transferred to a fresh vial followed by drying with a vacuum dryer at RT. The samples were reconstituted in 50 µL of 0.1% formic acid in deionized water, and peptide concentration was determined using the nanodrop 2000 (Thermo Scientific, San Jose, CA, USA).

Subsequently, the protein samples were subjected to liquid chromatography-electrospray ionization-mass spectrometry (LC-ESI-MS/MS), with two replicates per sample. One µg/µL of each sample was injected into the LC-ESI-MS/MS. Peptide fractionation and mass spectrometry analysis were performed using the liquid chromatography system Easy-nLC II (Thermo Scientific, San Jose, CA, USA) coupled with an LTQ-Orbitrap Velos Pro mass spectrometer (Thermo Scientific, San Jose, CA, USA). Fractionation of peptides was performed using Easy-Column C18-A2 (100 × 0.75 mm i.d., 3 μm; Thermo Scientific, San Jose, CA, USA) coupled with pre-column (Easy-Column, 20 × 0.1 mm i.d., 5 μm; Thermo Scientific, San Jose, CA, USA) at a flow rate of 0.3 μL/min and sample injection volume of 10 μL. The pre-column was equilibrated for 15 μL at a flow rate of 3 μL/min, whereas the analytical column was equilibrated for 4 μL at a flow rate of 0.3 μL/min. The running buffers used were (A) deionized distilled water with 0.1% formic acid and (B) ACN with 0.1% formic acid. The samples were eluted using a gradient of B from 5% to 100% in 100 min. Data were acquired with Xcalibur software version 2.1 (Thermo Scientific Co., San Jose, CA, USA) with a mass tolerance threshold of 5 ppm. The fragmentation technique used was collision-induced dissociation (CID) with a collision energy of 35. The database matching was performed using PEAKS software version 7.5 (Bioinformatics Solutions Inc., Waterloo, ON, Canada).

In the second peptide extraction method, vials containing microdissected FFPE tissue samples of the normal cervix (n = 3), SIL (n = 2), and SCC (n = 3) were covered with 20 µL 10 mM citric acid (pH 6.0), incubated for 60 min at 99 °C while shaking at 800 rpm using a Thermomixer (Eppendorf, Hamburg, Germany), and then allowed to cool at RT. The pH was adjusted to 7.4 using 500 mM ammonium bicarbonate. The samples were incubated with 0.1% *Rapi*Gest SF surfactant (Waters Corporation, USA) for 10 min while shaking at 800 rpm using a Thermomixer. Then, 1.2 µL10 mM DTT was added, and samples were incubated for 40 min at 56 °C with agitation at 500 rpm, followed by alkylation using 15 mM citric acid for 30 min, with agitation for 1 min at 500 rpm at RT in the dark. Five μL of 300 ng/μL modified porcine trypsin (Promega, Madison, WI) in 50 mM ammonium bicarbonate was added to the tissue pieces, and samples were incubated overnight at 37 °C. The double digestion was performed by adding 1.67 μL of 300 ng/μL trypsin in 50 mM ammonium bicarbonate and 28.33 μL of 80% ACN to the tissue pieces for 3 h at 37 °C. To stop the reaction, 1 µL of 10% TFA was added, followed by incubation for 45 mins at 37 °C while shaking at 600 rpm. Subsequently, the samples were centrifuged at 16,000× *g* for 10 min, and the supernatant was transferred to a fresh vial followed by drying with a vacuum dryer at room temperature. Reconstitution was performed using 9 μL of 2% acetonitrile, followed by desalting using Zip Tip Millipore (Overijse, Belgium). The samples were then sonicated for 10 min and transferred into the HPLC vials.

The samples were analyzed by LC-TIMS-TOF using an Ultimate 3000 (Thermo Scientific, San Jose, CA, USA) coupled to a timsTOF fleX (Bruker Daltonik, Billerica, MA, USA) with a CaptiveSpray Source. One µg of the sample was loaded onto a 25 cm (75 µm internal diameter) fused silica column that had been packed with 1.9 µm C18 particles. It was then heated to 50 °C using a Sonation Oven (Sonation lab solutions, Biberach an der Riss, Germany). Separation was performed using a 70 min linear gradient (3 to 20% acetonitrile in 0.1% formic acid) at a flow rate of 300 nL/min. TIMS-TOF analysis was performed in positive ion mode with a capillary voltage of 1900 V, drying gas flow rate of 3.0 L/min, and a drying gas temperature of 180 °C. A mass scan range of 100 to 1700 *m*/*z*, a 1/k0 range of 0.60 to 1.60 Vs/cm^2^ with a ramp time of 100 ms, a total cycle time of 1.06 s (9 PASEF MS/MS scans), target intensity of 11,000 with an intensity threshold of 2500, and an active exclusion of 0.90 min was utilized for DDA-PASEF analysis. A deflection 1 delta of 70.0 V, funnel 1 RF of 300.0 Vpp, funnel 2 RF of 200.0 Vpp, with CID energy of 0.0 eV, and multiple RF of 500.0 Vpp, were also utilized. TIMS-specific parameters were Δt1 of −20.0 V, Δt2 of −160 V, Δt3 of 110.0 V, Δt4 of 110.0 V, Δt5 of 0.0 V, Δt6 of 55.0 V, Funnel 1 RF of 450.0 Vpp, and collision cell in of 300.0 V. TOF calibration was performed through injection of 1 mM sodium formate, while TIMS calibration was performed using Agilent ESI-L Low Concentration tuning mix (Agilent Technologies, P.N. G1969-85000). The system was operated using Bruker Compass 4.1 for timsTOF Series, Control Version 6.2 (Build 1.2) (64-bit), and Compass HyStar 5.1 Version 5.1.8.1 supported with Thermo Scientific™ Dionex™ Chromeleon™ 7 Chromatography Data System Version 7.2.10 (23925).

### 2.3. Protein Identification

The LC-ESI-MS/MS results were analyzed separately for protein identification using the standard PEAKS workflow with the following parameters: *Homo sapiens* UniProt (http://www.uniprot.org/; accessed on 5 October 2021) reference proteome from the database released in 2020; trypsin for the enzyme;, carbamidomethylation as fixed post-translation modifications (PTM); and methionine oxidation as variable post-translation modifications (PTM). The error tolerance, parent ion, and fragment ion were set at 15 ppm and 0.8 Da, with a maximum of 2 missed cleavages per peptide and a maximum of 5 variable PTMs per peptide. The protein −10logP (threshold of ion score) was set to ≥20, Peptide Spectrum Match’s false discovery rate (FDR) was set to <0.1%, and the number of unique peptides was set to ≥1. The identified proteins were then pooled into three histological groups: normal cervix, SIL, and SCC. The results from both LC-ESI-MS/MS were then combined for further analysis. The proteins were classified manually by comparing the proteins present in either histological group (unique proteins), in two groups (normal vs. SIL, SIL vs. SCC), or in all three groups (overlapping proteins).

### 2.4. Protein Quantification

The raw files from mass spectrometry were processed with MaxQuant (version 2.0.3.0). The MS/MS spectra were matched to in silico-derived tryptic peptide fragment mass values from the *Homo sapiens* Uniprot human database and potential contaminants by the Andromeda search engine. The proteins were quantified using the following parameters: peptide mass limited to 4600 Da with a maximum of 2 missed cleavages and a maximum length of 7 amino acids. Carbamidomethylation was set for fixed post-translation modifications (PTM), methionine oxidation, and acetylation of protein N-terminal as variable post-translation modifications (PTM). The main search, first search, and MS/MS tolerance were set at 6 ppm, 20 ppm, and 20 ppm respectively. Label-free quantification (LFQ) was performed using classic normalization and a minimum ratio count of 2. A reverse sequence library was generated to control the false discovery rate at <1% for protein group identifications. The data from MaxQuant analysis were further analyzed using Perseus (version 1.6. 14.0). Data were filtered to remove potential contaminants, reverse hits, and protein groups “only identified by site”. The protein intensities were Log(2x) transformed and annotated according to category (normal cervix, SIL and SCC). T-tests were used to compare the differences between the groups. Cutoff values of 1.5 and −1.5 was used for differentially expressed proteins. The combined proteins identified from both mass spectrometry instruments were used to determine the differentially expressed proteins between the histological groups.

### 2.5. Bioinformatics Analysis

Identified proteins were classified using DAVID (Functional Annotation Bioinformatics Microarray Analysis) version 6.8 (released 19 February 2022) at https://david.ncifcrf.gov/; accessed on 8 November 2021. Based on the protein ID, the molecular functions and biological processes were generated using DAVID software. The pathway enrichment analysis and protein-protein interaction were generated using ConsensusPathDB and STRING tools respectively. Although our results revealed many highly expressed proteins, such as histones, collagens, desmin, and vimentin, they were not chosen for subsequent immunohistochemical evaluation as they are ubiquitously expressed in most human cells. Using data-independent acquisition nano-flow liquid chromatography-mass spectrometry on FFPE tissue samples, two proteins, annexin A2 (ANXA2) and cornulin (CRNN), were selected for validation in a larger cohort by immunohistochemistry (IHC). Other differentially expressed proteins, such as histones, collagen, and vimentin were not chosen as they are ubiquitously expressed in most cells. Annexin A2 was found to be downregulated in the transformation from the normal cervix to SIL and upregulated in the progression from SIL to SCC in the label-free quantification using MaxQuant and Perseus software. ANXA2 has also been shown to be a useful biomarker in other female reproductive cancers [32,33]. In contrast, cornulin showed a decreasing trend in expression using the label-free quantification method, being highest in the normal cervix and lowest in SCC. Furthermore, cornulin was reported to be downregulated in other squamous cell carcinomas of the esophagus, head, and neck [31,34,35].

### 2.6. Immunohistochemistry of Annexin A2 (ANXA2) and Cornulin (CRNN) Proteins

Immunohistochemistry (IHC) for each protein was performed on two commercial tissue microarrays (TMA) (BB10011 and CR1101, Biomax Inc, Derwood, Maryland) comprising 10 cases of normal cervixes, 21 SIL, and 101 squamous carcinoma cases. The TMAs had accompanying data on pathology diagnosis, SIL grade (Bethesda Classification System), and the International Federation of Gynecology and Obstetrics (FIGO) staging for cervical cancer. IHC was performed using the horseradish peroxidase polymer method from EnVision ™ FLEX/HRP (Dako, Via Real Carpinteria, CA, USA) as mentioned in 2.2 with Annexin A2 (ANXA2) rabbit monoclonal antibody (1:500 dilution; cat. no. 8235S, Cell Signaling Technology, Danvers, MA, USA) or Cornulin (CRNN) polyclonal antibody (1:500 dilution; cat. no. 11799-1-AP, Proteintech, Rosemont, IL, USA) as primary antibody. The mounted slides were scanned with MoticEasyScan Pro (Motic, Kowloon, Hong Kong). The digitally scanned slides were analyzed using QuPath software. Brown staining signified positive protein expression. The percentage of positive cells was scored from 0 to 4 (0 = 1% positive cells; 1 = 1 to 25% positive cells; 2 = 25 to 50% positive cells; 3 = 25 to 50% positive cells; 4 = more than 75% positive cells) and the staining intensity was scored from 0 to 3 (0 = no staining; 1 = weak staining; 2 = moderate staining; 3 = strong staining). The histoscore was calculated by multiplying the percentage of the positivity score and staining intensity score, as shown in Supplementary Table S1. The final histoscore was scored from 0 to 12 (0 = negative; 1 to 3 = low expression; 4 to 12 = high expression). Colorectal cancer tissue was used as a positive control, as suggested in the datasheet, where brown staining was observed in the cytoplasm and membrane of cancer cells. Primary antibodies were omitted in the negative control.

### 2.7. Statistical Analysis

The association between ANXA2 and CRNN histoscore and histological groups, as well as clinicopathological parameters, were analyzed by the Pearson chi-square test; the significance was set at a *p*-value ≤ 0.05, using the IBM SPSS v28.0.1 software package for Windows.

## 3. Results

### 3.1. Protein Identification

**Figure 1:**
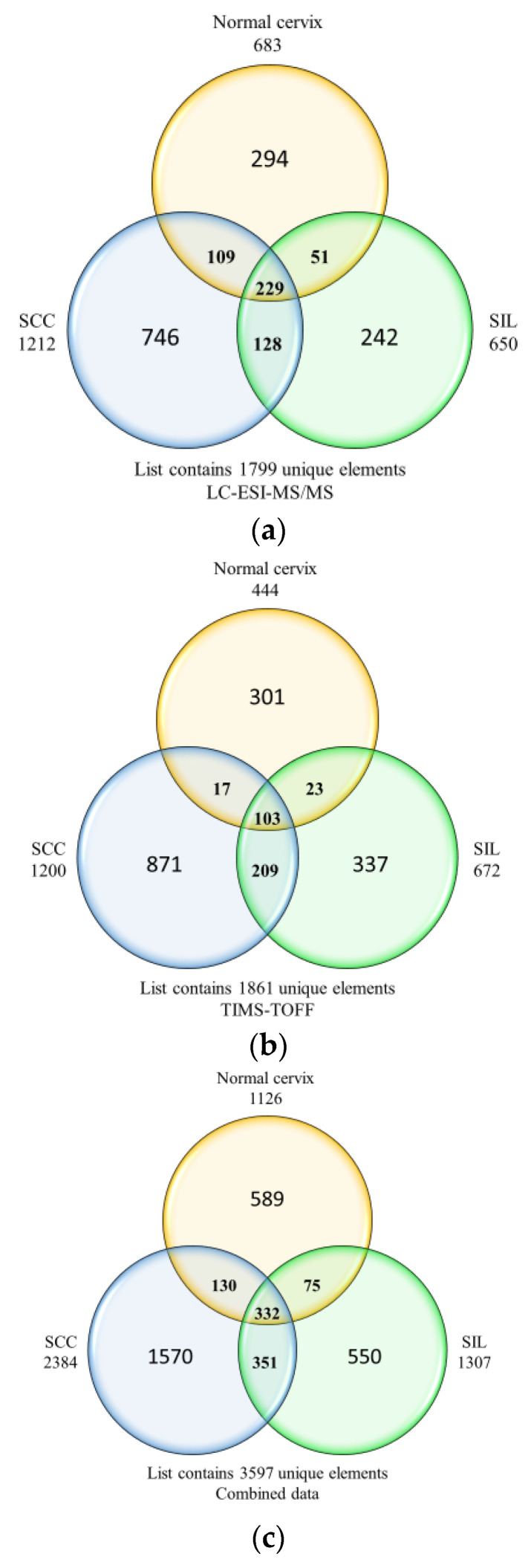
Venn diagram depicting the unique and overlapping proteins in the normal cervix, SIL, and SCC groups from (**a**) LC-ESI-MS/MS (**b**) timsTOF MS and (**c**) combined data. The protein is considered significant when −10logP is ≥20 and the unique peptide is ≥1.

Two mass spectrometry instruments employed in this study are complementary ionization techniques, and the use of both systems aids in a greater identification of peptides. The proteins were analyzed according to the three histological groups of normal cervices, SIL, and SCC, fulfilling the criteria of −10logP ≥20 and unique peptides ≥1. A total of 1799 and 1861 proteins were identified from the LC-ESI-MS/MS and LC-ESI-timsTOF MS, respectively. The unique proteins for each histological group and overlapping proteins identified for both mass spectrometry methods are displayed in Venn diagrams in Figure 1, while Supplementary Table S2 shows the combined list of proteins identified from the LC-ESI-MS/MS and timsTOF instrument. The proteins identified from both mass spectrometries showed similarities of 88.6% for normal cervix, 92.2% for SIL, and 93.4% for the SCC group. As such, there is a good concordance in the proteins extracted from FFPE tissues between the orbitrap and timsTOF instrument.

### 3.2. Differentially Expressed Proteins

Protein quantification was performed to identify the differentially expressed proteins in the progression from the normal cervix to SIL and SCC. The quantified proteins from orbitrap and timsTOF results were combined by including the proteins of similar accession numbers in a group. The number of differentially expressed proteins identified in the comparison groups are listed in Table 1, and they satisfy the criteria of fold change ≤ −1.5 and *p*-value < 0.05. In SIL, 39 proteins were downregulated compared to the normal cervix, where there were no upregulated proteins. Overall, the histone H2A proteins were highly downregulated (−4.70-fold change) followed by hemoglobin subunit alpha (−3.36-fold change), while cornulin showed a −1.50-fold change, as shown in Supplementary Table S3. A graph of differentially expressed proteins is shown in Supplementary Figure S1. In the SCC vs. SIL group, we discovered 51 upregulated proteins and an absence of any downregulated proteins. Interestingly, histone H2A was the most upregulated protein, with a fold change of 15.94 followed by actin (9.84-fold change) and histone H2B (9.18-fold change). Annexin A2 revealed a 2.30-fold change. In the SCC vs. normal cervix group, the results demonstrated 49 upregulated proteins and 4 downregulated proteins. Among the upregulated proteins, actin cytoplasmic 2 was the most upregulated protein (13.29-fold change), followed by histones 2A (11.24-fold change). All the upregulated proteins in the SCC vs. normal cervix groups are from the actin and histone families. The topmost downregulated protein was collagen alpha-3(VI) chain (−2.96-fold change). Out of four downregulated proteins, three were from the collagen family. Interestingly, cornulin was also downregulated in the SCC vs. normal cervix group with (−2.54-fold change).

### 3.3. Gene Ontology, Pathways, and Protein-Protein Interaction Networks in Cervical Cancer Development

The molecular functions and biological processes of the differentially expressed proteins between the histological groups were analyzed using DAVID software, while ConsensusPathDB was used for the pathway enrichment analysis and STRING for protein-protein interaction networks.

#### 3.3.1. SIL vs. Normal Cervix

**Figure 2:**
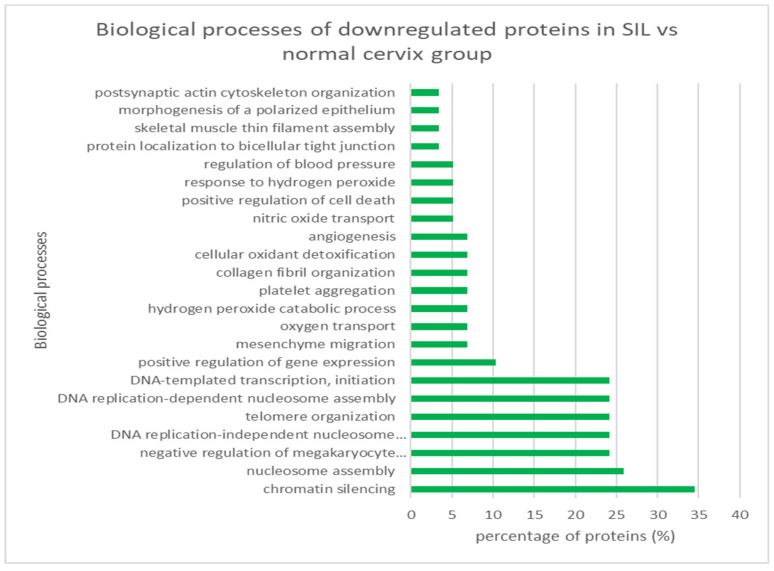
Classification of biological processes of downregulated proteins in SIL vs. normal cervix group. The percentage reflects the number of proteins involved. The gene ontology classifications were generated using DAVID version 6.8 (released 19 February 2022). Protein–protein interaction network analysis was performed with STRING.

In SIL, there were 39 downregulated differentially expressed proteins compared to the normal cervix. When categorized according to molecular functions, the largest categories were associated with protein heterodimerization activity and DNA binding, with 58% proteins involved and a *p*-value <0.05 (Supplementary Figure S2). Of the total differentially expressed proteins, 57.9% were involved in the different binding processes, including DNA, protein domain-specific, RNA, haptoglobin, organic acid, oxygen, collagen, enzyme, heme, hemoglobin alpha, and platelet-derived growth factor binding. The results demonstrated 24 biological processes, with chromatin silencing as the most common and comprising 34.5% of proteins. The other notable biological process was the nucleosome assembly (Figure 2). The protein–protein interaction networks indicated that the proteins are at least partially biologically connected as a group (Supplementary Figure S3). ANXA2 is also co-expressed with actin cytoplasmic 1, actin cytoplasmic 2, filamin A, and vimentin. In contrast, CRNN did not interact or co-express with other proteins. The results demonstrated 25 pathways according to the pathway analysis (Table 2), with the most common being the signaling events mediated by HDAC Class III with 35.9% proteins, hemoglobins chaperone (23.1%), and neutrophil extracellular trap formation (19.5%).

#### 3.3.2. SCC vs. SIL

**Figure 3:**
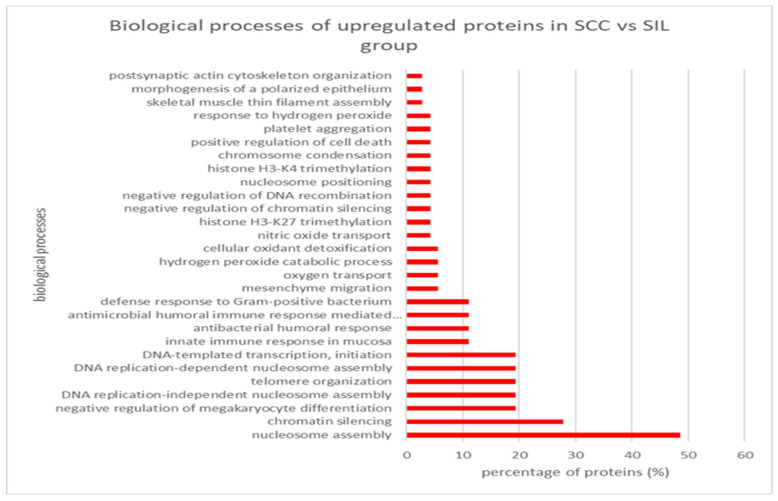
Classification ofbiological processes of upregulated proteins in the SCC vs. SIL group. The percentage reflects the number of proteins involved. The gene ontology classifications were generated using DAVID version 6.8 (released 19 February 2022). Protein–protein interaction networks were performed using STRING analysis.

A total of 51 upregulated differentially expressed proteins were found in SCC compared to SIL groups. The largest molecular function categories were associated with protein binding, protein heterodimerization activity, and DNA binding, with 77.8%, 70.8%, and 70.8% proteins involved, respectively, with a *p*-value <0.05 (Supplementary Figure S4). The majority (70.6%) of the differentially expressed proteins were involved in the binding process, similar to SIL vs. normal cervix comparison. The results demonstrated 28 biological processes, the most common being nucleosome assembly, comprising 48.6% of proteins, and chromatin silencing, with 27.8% of proteins involved (Figure 3). In the protein–protein interaction analysis, ANXA2 is co-expressed with actin cytoplasmic 1 and actin cytoplasmic 2 (Supplementary Figure S5). Based on the pathway analysis, we found a total of 8 pathways (Table 3), with the most common being the signaling events mediated by HDAC Class III (38.5% of proteins), neutrophil extracellular trap formation (28.9%), and hemoglobins chaperone (23.1%).

#### 3.3.3. SCC vs. Normal Cervix

**Figure 4:**
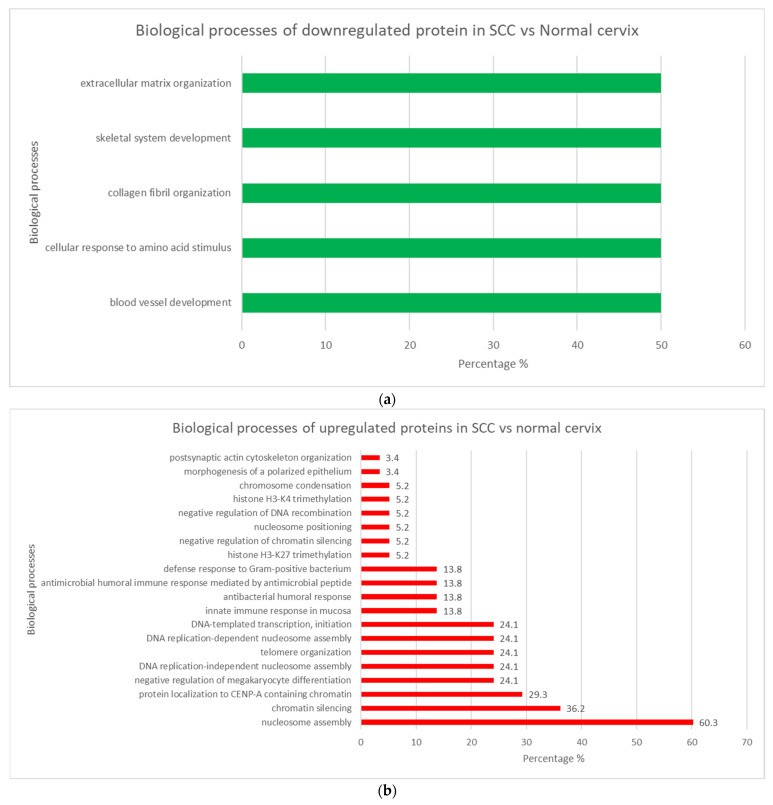
Classification of biological processes of (**a**) upregulated and (**b**) downregulated proteins in SCC vs. normal cervix group. The percentage reflects the number of proteins involved in each biological process. The gene ontology classifications were generated using DAVID version 6.8 (released 19 February 2022).

In the upregulated proteins in SCC vs. normal cervix, the largest categories were associated with structural constituent of chromatin with 93.1% proteins involved and a *p*-value <0.05. Protein heterodimerization activity and DNA binding are the second most involved molecular function, with 89.7%. The molecular functions of the upregulated proteins in SCC vs. normal cervix group are shown in Supplementary Figure S6. Among the upregulated proteins in the SCC vs. normal group, the most common biological process was nucleosome assembly (60.3%), followed by chromatin silencing (36.2%). The other notable biological process was the protein localization to CENP-A containing chromatin (29.3% proteins). Figure 4a illustrates the biological processes involved in the upregulated proteins of SCC vs. normal cervix group. The protein–protein interaction network in the upregulated proteins in SCC vs. normal cervix group shows histones as the most abundant protein cluster. The interactions are clustered into three groups: histone 2A, histone 2B, and actin. The protein–protein interactions of the upregulated proteins in SCC vs. normal cervix group are illustrated in Supplementary Figure S7. According to the pathway enrichment analysis, neutrophil extracellular trap formation (28.9%) was the most common signaling pathway involved in the upregulated proteins of SCC vs. normal cervix. Viral carcinogenesis (15.7%) and necroptosis (4.0%) were also found in the upregulated proteins of SCC vs. normal cervix group. The summary of pathways involved in the upregulated proteins of SCC vs. normal cervix is shown in Table 4.

There were only four molecular functions involved in the downregulated proteins, including the extracellular matrix structural constituent conferring tensile strength (75%), platelet-derived growth factor binding (50%), protease binding (50%), and extracellular matrix structural constituent (50%). The molecular functions of the downregulated proteins in SCC vs. normal cervix group are shown in Supplementary Figure S8. There were five biological processes in the downregulated proteins of SCC vs. normal cervix group, including blood vessel development, cellular response to amino acid stimulus, collagen fibril organization, skeletal system development, and extracellular matrix organization. Figure 4b illustrates the biological processes involved in the upregulated proteins of SCC vs. normal cervix group. The protein–protein interactions in the downregulated proteins show interactions among the collagen, and cornulin did not interact or co-express with other proteins. The protein–protein interaction network of downregulated proteins in SCC vs. normal cervix group is illustrated in Supplementary Figure S9. VEGFR3 signaling in lymphatic endothelium (8.0%) was the most common pathway involved in downregulated proteins of the SCC vs. normal cervix group. The other interesting pathways include proteoglycans in cancer (1.0%), human papillomavirus infection (0.9%), and PI3K-Akt signaling pathway (0.8%). The summary of pathways involved in the downregulated proteins of SCC vs. normal cervix is shown in Table 4.

### 3.4. Annexin A2 (ANXA2) and Cornulin (CRNN) Immunohistochemistry

The annexin A2 protein expression was scored in 125 cases on 2 tissue microarrays that comprised 8 normal cervixes, 21 SIL, and 96 SCC cases; 9 tissue cores were excluded due to the absence of squamous cells. The protein was localized in the cytoplasm and membrane of squamous cells. The expression is considered negative if the histoscore is zero, low when the histoscore is 1–3/12, and high when the histoscore is ≥4/12. As stated in Table 5, the results showed no statistically significant difference in annexin A2 expression across the different histological groups (*p* = 0.35). Furthermore, our analysis did not reveal any significant correlation between its expression and the severity of SIL grade or SCC stage. However, there was a significant association between annexin A2 and the age of the subjects (*p* = 0.04), as shown in Table 6.

**Figure 5:**
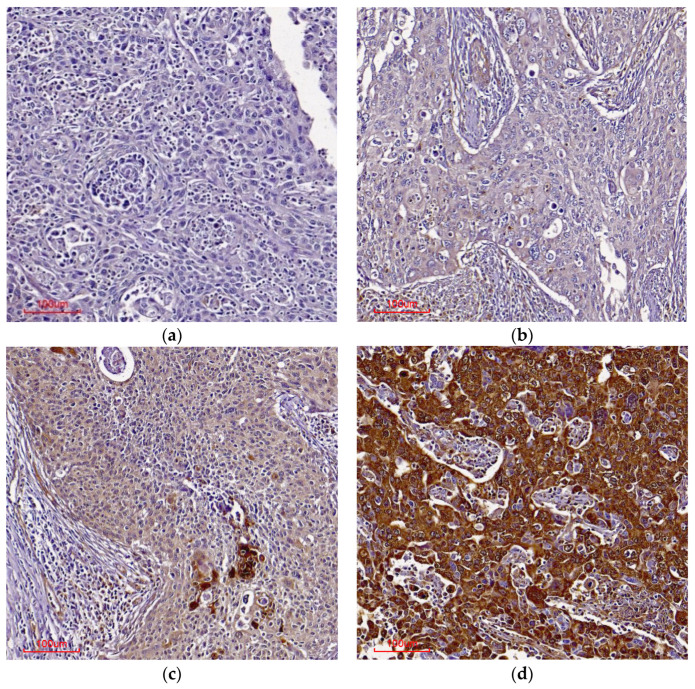
Immunohistochemical staining pattern in cervical tissue cores using CRNN antibody. (**a**) Absent staining in squamous cell carcinoma (SCC) cells. (**b**) Weak intensity cytoplasmic stain in SCC cells (**c**) Moderate intensity staining in the cytoplasm of SCC cells. (**d**) Strong intensity staining in the cytoplasm and membrane of SCC cells. Scale bar: 100 µm, magnification: 100×.

The cornulin histoscore was performed on 127 cases that comprised 8 normal cervixes, 21 SIL, and 97 SCC cases, with the exclusion of 8 tissue cores that did not contain squamous epithelium. The protein was located in squamous cell cytoplasm and exhibited a statistically significant difference across the histological groups (*p* = 0.004). In particular, there was a significant difference in expression levels between SIL and normal cervix (*p* = 0.05), as well as SCC and normal cervix (*p* = 0.01), with a decreasing trend observed from normal cervix to SCC (Table 5). Furthermore, cornulin expression was significantly associated with SIL grade (*p* = 0.01), but not with subject’s age or SCC stage (Table 6). Representative immunohistochemical staining patterns for cornulin are provided in Figure 5.

## 4. Discussion

Cervical carcinomas arise from the normal cervical epithelium through the progressive development of low- and high-grade cervical intraepithelial lesions [36]. The HPV viral oncogene products alter the host genome, proteome, and intracellular signaling network of the cervical epithelium, promoting oncogenesis. Protein biomarkers in biological samples are dynamic and change in response to environmental conditions, genetics, diet, obesity, chronic diseases such as diabetes, and therapeutic intervention. Proteomics data can be applied to the entire spectrum of health or disease progression, from early screening/diagnosis to drug discovery and precision therapy [37].

Proteomics remains the method of choice in cancer research to identify proteins associated with cancer development and progression. It also offers the discovery of novel biomarkers and therapeutic targets through insight into the underlying molecular mechanisms. Many proteomics studies have been conducted on cervical cancer; however, limited reports focus on the development of the disease. Our study compared the protein profiles of the normal cervix, precancerous SIL, and cervical cancer tissues derived from FFPE samples, using two sensitive high throughput instruments LTQ-Orbitrap, timsTOF MS, PEAKS software, and further analysis with relevant bioinformatics tools.

The comparison of mass spectrometry performance is a controversial topic as the specifications of instruments differ depending on the type of application and the experimental setting. The results obtained from the two mass spectrometry instruments used in this study show good concordance with more than 85% similarities for the number of proteins identified in the three histological groups. While FFPE tissues provide invaluable resource material for proteomics studies, the cross-links between proteins and chemical modifications associated with FFPE treatment are complex, affecting protein yield and quality. Although there is no consistency in the technique used for protein extraction, the potential for protein recovery from FFPE tissues has increased [38]. Our study proves that both urea lysis buffer and *Rapi*Gest SF surfactant are useful methods for protein extraction from FFPE tissues.

Various biological processes and pathways are perturbed in the development of cervical cancer. In the transition from the normal cervix to SIL, our results showed that all 39 differentially expressed proteins were downregulated with a significant number of proteins related to the binding process and comprising histones (Supplementary Figure S2). It is well known that histone post-translational modification regulates a wide range of important biological processes, usually through chromatin modification that promotes or inhibits the expression or repression of target genes. Histone modification proteins include histone families H2A, H2B, H3, and H4; two H2A/H2B heterodimers; and one H3/H4 tetramer coupled with DNA [39]. Histone H2A and H2B play vital roles in chromatin functions, such as transcription, DNA replication, and DNA repair [40], supporting the role of the binding process in our findings. Protein–protein interactions (PPIs) play vital roles in fundamental processes in living cells. PPIs are involved in multi-functions, including modifying the kinetic properties of enzymes, catalyzing metabolic reactions, activating, or suppressing a protein, changing the specificity of a protein, regulating upstream and downstream levels, and transporting molecules [41]. The signaling events mediated by HDAC Class III were the most common pathway involved in our study (Table 3). Histone deacetylases (HDAC) are enzymes that remove acetyl groups from histone lysine residues [42]. The HDAC class III, also known as the Sirtuins (SIRT1-7), can deacetylate a combination of histones and nonhistone proteins [42]. Several other interesting pathways include necroptosis, viral carcinogenesis, proteoglycans in cancer, PI3K-Akt signaling pathway, and caspase cascade in apoptosis. Necroptosis is implicated in the regulation of cancer biology, including oncogenesis, cancer metastasis, cancer immunity, and cancer subtypes [43,44]. Some of the factors that may trigger necroptosis are DNA binding, adhesion, dependence receptors, immunological responses, infections, and different medications [45,46]. Viral carcinogenesis is closely related to the HPV oncogenes E6 and E7, in which an increase in viral oncogene expression in HPV-positive lesions of patients is associated with progression to cervical cancer [47]. The suppression of annexin A2 (−1.83-fold change) in our study may affect the entry of HPV into target cells, preventing persistent HPV infection. Dziduszko and Ozbun (2013) found that the ANXA2 core domain is involved in the binding of HPV16 at the cell surface. The interaction of viruses with ANXA2 at the membrane as well as within endocytic vesicles suggests that ANXA2 plays an active and direct role in viral entry [48]. ANXA2 core domain interacts with F-actin and a few proteins, such as S100 and histones. This is further supported by the protein–protein interaction analysis in our study (Supplementary Figure S5). We discovered that, among the downregulated proteins, the ECM receptor interactions pathway appeared to be crucial; it includes collagen, actin, filamin A, and prolargin. Downregulation of collagen promotes the invasion of cancer into the baseline membranes and stroma. Collagen can promote additional signaling pathways in cancer cells to exert numerous functions such as the caspase-3/PI3K/AKT pathways that can inhibit cell apoptosis in cervical cancer tissues [49]. PI3K is a downregulator of the Ras signaling pathway and is involved in receptor signal transduction. The PI3K pathway regulates various cellular and molecular functions essential for tumor initiation, invasion, growth, proliferation, metastasis, and apoptosis [50,51,52]. Signaling proteins bind to the lipid products of PI3K and are localized on the cell membrane to activate cell growth and survival pathways [53]. Bahrami et al. reported that the PI3K pathway plays a crucial role in cervical cancer for prognostic purposes [50].

Interestingly, all the differentially expressed proteins were found to be upregulated in the progression from SIL to SCC (Table 1). Furthermore, many proteins and pathways discovered were similar to the normal cervix vs. SIL comparison. The proteins may be similar due to the protein–protein interactions and the regulation at the gene level. Most of the proteins here were also involved in the binding process (DNA, RNA, protein binding), with histones H2A and H2B as the predominant protein group. Histones H2A and H2B are also involved in nucleosome assembly in the biological process. Other biological processes which involve histones include nucleosome positioning, histone H3-K27 trimethylation, and histone H3-K4 trimethylation. Nucleosome position and histone marks are critical for distinguishing regulatory elements, such as promoters, enhancers, and repressed regions. Murakami et al., (2021) reported that the nucleosome positioning in the chromatin organization of the HPV16 and HPV18 long control region and E6 is an important regulatory event during the viral life cycle that is dependent on the status of host cell differentiation [54]. As shown in this study, proteins such as histones, actins, and tubulins are involved in the signaling events mediated by the HDAC class III pathway. The interactions among the proteins may be the cause of the changes in the expression level of the proteins from one stage to another. The neutrophil extracellular trap formation pathway is also implicated. In the inflammatory environment, these neutrophil-derived extracellular structures consist of decondensed DNA complexed with citrullinated histones (H3Cit) and numerous neutrophil granule proteins such as myeloperoxidase (MPO) and neutrophil elastase (NE) [55,56]. The neutrophil extracellular trap formation is involved in tumor progression and is closely related to tumor proliferation, metastasis, and thrombosis [57]. Our results also revealed an upregulation of necroptosis and viral carcinogenesis due to the presence of histones H2A and H2B.

When compared to the normal cervix, a total of 53 proteins were differentially expressed in SCC, with 49 upregulated proteins and 4 downregulated proteins (Table 1). Histones were predominantly seen in the upregulated proteins group, whereas collagen was the main downregulated protein in the SCC vs. normal group (Supplementary Table S3). Based on the molecular function, most of the upregulated proteins are involved in binding processes, such as DNA, RNA, protein, and enzyme binding. The structural constituent of chromatin (93.1%) was the most common molecular function in the upregulated proteins (Supplementary Figure S6). According to biological process analysis, there were a significant number of proteins involved in the nucleosome assembly (60.3%) in upregulated proteins. This may be due to the differentially expressed histones with fold change of 11.2 (Supplementary Table S3). In this study, the pathway analysis revealed that most of the proteins are involved in the neutrophil extracellular trap formation pathway for the upregulated proteins (Table 4). Neutrophil extracellular traps (NETs) are composed of DNA fibers, histones, granular content, and antimicrobial proteins that help to trap and kill invasive bacteria [58]. NETs play a complex and important role in cancer progression, metastasis, angiogenesis, cancer-associated thrombosis, and therapy. Yan et al. (2021) reported that combining clinical stage assessment with NET assessment may improve prognostic stratification, and may lead to the development of new therapeutic strategies for cervical cancer [59].

In comparing SCC to the normal cervix, the downregulated proteins showed the extracellular matrix structural constituent conferring tensile strength as the most common molecular function (Supplementary Figure S8). There are a total of four molecular functions which are correlated to collagen. In addition, the biological processes are also mostly associated to collagen in the downregulated proteins. Downregulation of collagen promotes the invasion of cancer into the baseline membranes and stroma. Collagen can promote additional signaling pathways in cancer cells to exert numerous functions, such as the caspase-3/PI3K/AKT pathways that can inhibit cell apoptosis in cervical cancer tissues [49]. Interestingly, VEGFR3 signaling in the lymphatic endothelium pathway was involved in the SCC vs. normal cervix groups in this study. In cervical cancer, the angiogenic process is associated with VEGF expression, which is related to the severity of precursor lesions and invasive disease [60,61]. However, in this study, the VEGF pathway was one of the downregulated proteins in the normal vs. SCC group. The results also revealed a few other pathways in the downregulated proteins of the SCC vs. normal cervix group, including the PI3 kinase pathway.

**Figure 6:**
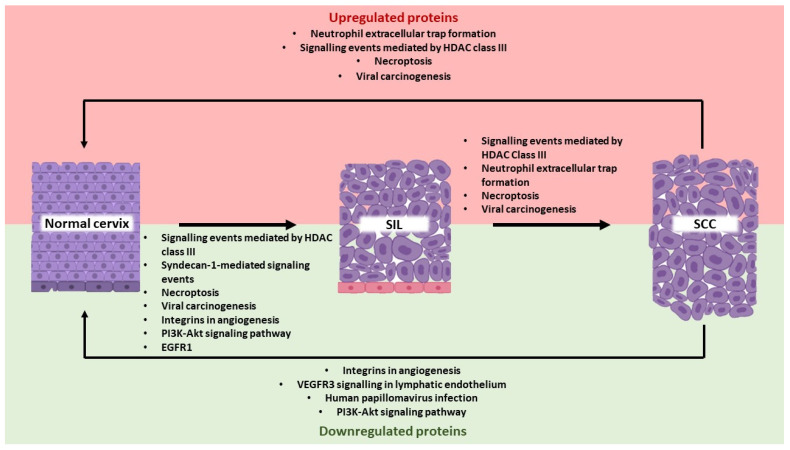
Summary of the pathways involved in the development of cervical cancer. The figure illustrates the upregulated (pink region) and downregulated pathways (green region) involved in cervical carcinogenesis, from normal cervix to SIL and SCC. The main signaling events mediated by HDAC Class III, necroptosis, and viral carcinogenesis are downregulated in the transformation from the normal cervix to SIL, which then become upregulated during the progression from SIL to SCC. The same pathways are also found to be upregulated in SCC compared to normal cervix, suggesting that their activation pathways trigger cancer formation. In contrast, the PI3K-Akt signaling pathway and integrins in angiogenesis are downregulated in the transition from normal cervix to SIL, as well as in SCC compared to normal cervix, which are involved in cell proliferation, growth and metastasis.

There are a few parallel pathways involved in the development of cervical cancer from a normal cervix. The similarities may be due to ubiquitous proteins, including histones, collagen, and actin, that are found in more than one histological group. Some of the pathways are switched on and off depending on the expression of the proteins. A summary of the critical pathways involved is shown in Figure 6.

The progression of HPV-infected cervical tissues into the precancerous stage (SIL) and SCC offers an advantage to studying the roles of ANXA2 and CRNN molecules in cervical carcinogenesis. Several studies suggest that these proteins are involved in the tumorigenesis of various cancers. ANXA2 stimulates cell invasion in breast, brain, liver, and pancreatic cancers, and improves cell motility and adhesion in prostate and hepatocellular carcinoma cells [62,63,64]. The interaction between ANXA2 and its binding proteins plays a significant role in the tumor microenvironment by promoting cancer metastasis in ovarian cancer [65]. The suppression of annexin A2 (−1.83-fold change) in this study may affect the entry of HPV into target cells and persistent HPV infection. Dziduszko and Ozbun (2013) found that the annexin A2 core domain is involved in the binding of HPV16 at the cell surface. The interaction of viruses with annexin A2 at the membrane as well as within endocytic vesicles suggests that annexin A2 plays an active and direct role in viral entry [48]. The annexin A2 core domain interacts with F-actin as well as proteins such as S100 and histones. This is further supported by the protein–protein interaction analysis in our study (Supplementary Figure S3). While protein quantification from LC-MS results indicated downregulation of ANXA2 in SIL vs. normal cervix (−1.83-fold change) and upregulation in SCC vs. SIL (2.3-fold change), these findings were not reflected in immunohistochemistry results.

**Figure 7:**
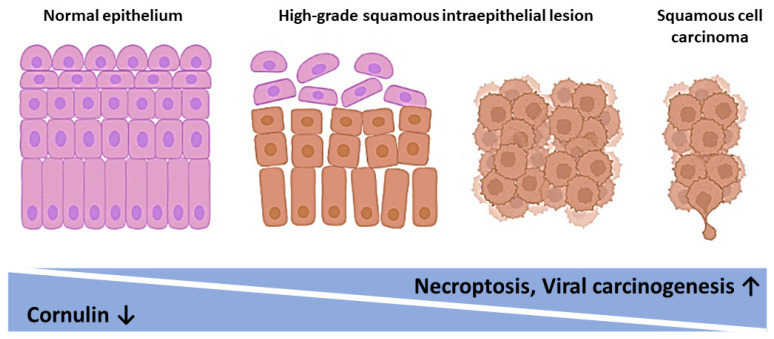
Cornulin expression and key pathways involved in cervical cancer development. As cornulin expression decreases, necroptosis and viral carcinogenesis pathways appear to escalate, indicating cornulin as a plausible tumor suppressor. (Created using Biorender software).

The precise role of ANXA2 in cervical cancer is poorly understood, as indicated by conflicting reports on its protein expression. Choi et al. reported a decreased expression of ANXA2 in cervical cancer tissues compared to normal tissues [66] while Zhe Wang et al. reported a significantly higher ANXA2 expression in cancer compared with normal cervical tissues [67]. In the ANXA2 immunohistochemical evaluation of 125 tissue cores in our study, we found a high histoscore in more than 90% of the total number of tissue cores in each histological group, resulting in an insignificant statistical difference between the groups (Table 5). There was no association between the expression of ANXA2 to the SIL grade or SCC stage (Table 6). Our study provides evidence for the involvement of ANXA2 in cervical carcinogenesis. We found that ANXA2 is overexpressed in SIL and SCC, where it activates the PI3K/AKT pathway, leading to increased cell proliferation and growth, as well as inhibition of apoptosis. Furthermore, ANXA2 promotes cancer cell invasion into the squamous epithelium basement membrane and underlying connective tissue by interacting with intracellular proteins that are essential for metastasis. Despite its significant role in cancer progression, the high immunohistochemical expression of ANXA2 in normal cervix remains poorly understood. Further research is needed to elucidate the underlying mechanisms and potential implications of the cervix in health and disease and to explore therapeutic inhibitors against ANXA2. The results of this study showed a decreasing trend in CRNN protein expression with the highest levels observed in the normal cervix group and the lowest levels in the SCC group, as evidenced by both the LC-MS and immunohistochemistry results. These findings are comparable with another study by Arnouk et al., (2009) who found a decline in CRNN expression in SIL and a further decrease in cancer [23]. Cornulin gene expression was also found to be downregulated in a study comparing SCC to the normal cervix (−31.1-fold-change) [68]. Cornulin is a “fused gene” protein that binds calcium and is upregulated in response to deoxycholate-induced stress [30,69]. It is generally expressed late in epidermal differentiation, but its function is uncertain. Cornulin has been studied in inflammatory diseases and may be involved in the pathogenesis of psoriasis. However, its precise role has not been elucidated. [70]. Cornulin has been proposed as a tumor suppressor gene, controlling pathways involved in cancer cell proliferation, migration, invasion, and apoptosis, however, its mechanism of action is still unknown [31]. In head and neck squamous cell cancer, the downregulation of cornulin may cause the squamous epithelia to be more susceptible to infection and eventually lead to carcinogenesis [71]. When addressing the association between cornulin expression and carcinogenesis, Imai et al. reported that cornulin functions as a tumor-suppressing element by arresting the cell cycle at the G1/S phase through downregulation of cyclin D1 [72]. Xiao et al., reported that cornulin could significantly distinguish healthy controls from dysplasia and oral cancer [73]. Though little is known about the molecular basis of cornulin downregulation in oral squamous cell carcinoma, no correlation with structural anomalies or epigenetic changes, such as promoter methylation, has been found [74]. Our study observed that cornulin expression reduced as cells became less differentiated in the dysplastic and cancer phases. This was evidenced by a significant association between cornulin and SIL grade (*p* = 0.010) with a high histoscore in 8 out of 10 SIL grade 1 cases and 0 of 7 SIL grade 3 cases (Table 6). It was also not surprising that 21/54 cases (38.8%) of SCC Stage I cases exhibited a high histoscore compared to only 1 out of 5 cases (25%) in SCC Stage III, though this was statistically insignificant. Cornulin is a protein that is specific to squamous cells, and our study found that it is highly expressed in normal cervical cells. Our results suggest that cornulin may have a role as a potential tumor suppressor, as its expression decreases as the cells progress from normal to SIL and SCC. Furthermore, it is known that cancer cells can acquire epigenetic changes that lead to silencing of genes and downregulation of proteins. Finally, cornulin may be a potential target for therapeutic interventions in cervical cancer. A summary of cornulin expression and the key pathways involved in cervical cancer development is illustrated in Figure 7.

As proteins are built through mRNA transcription, based on its nucleotide sequence, we were compelled to compare the current protein profile to a transcriptomics study published by our research group using a different set of FFPE cervical tissue samples grouped into the normal cervix, SIL, and SCC [68]. The proteome results of the present study appear concordant with the transcriptomics data whereby both studies showed downregulation of the ECM, collagen, apoptosis, and PI3K pathway in the SIL group compared to the normal cervix. Balasubramaniam et al. (2022) reported that the MMP9 gene was upregulated and promoted collagen breakdown between epithelial cells and basement membrane, causing cell invasion into deeper tissue. The breakdown of collagen leads to the migration and invasion of dysplastic cells [75]. We also found the ECM organization in molecular function and pathway to be downregulated in the normal cervix vs. SIL group in the present study, supporting the transcriptomics study.

## 5. Conclusions

In HPV-associated cervical carcinogenesis, our results showed 39 downregulated proteins in the transition from the normal cervix to SIL and 51 upregulated proteins in the progression from SIL to SCC. Most proteins interact with one or more specific sites of another molecule in a selective, non-covalent, and stoichiometric manner. In addition, proteins related to the PI3 kinase pathway play a critical role in initiating the neoplastic transformation from the normal cervix to SIL, whereas the necroptosis and viral carcinogenesis pathways are crucial for sustaining cell invasion, apoptosis, and metastasis when SIL progresses to SCC. Annexin A2 did not prove to be a good biomarker as it was highly expressed in all three histological groups. Cornulin may be a potential biomarker for the risk of disease progression, whereby it becomes more downregulated as the disease progresses. The results support its role as a tumor suppressor gene, controlling pathways involved in cancer cell proliferation, migration, invasion, and apoptosis. Further studies on cornulin as a biomarker for disease progression are warranted.

As with most studies, the design of the current study is subject to limitations. The functions of CRNN have not been studied using cervical squamous cell carcinoma and endocervical adenocarcinoma (CESC) cell lines. The downstream analysis of the functions of CRNN protein would provide a better understanding of the underlying mechanisms in cervical cancer.

## Figures and Tables

**Table 1:** Number of differentially expressed proteins in the histological.

Histological Groups	Total Number of Differentially Expressed Proteins	Number of Upregulated Proteins	Number of Downregulated Proteins
**SIL vs. normal cervix**	39	0	39
**SCC vs. SIL**	51	51	0
**SCC vs. normal cervix**	53	49	4

**Table 2:** Pathways involved in downregulated proteins in SIL vs. Normal cervix group using ConsensusPathDB software.

Pathways	Percentage of Proteins (%)	*p*-Value	Pathway Source
Signaling events mediated by HDAC class III	35.90%	1.48 × 10^−24^	PID
Hemoglobins chaperone	23.10%	2.00 × 10^−5^	BioCarta
Neutrophil extracellular trap formation	19.50%	8.31 × 10^−56^	KEGG
Necroptosis	13.20%	1.27 × 10^−26^	KEGG
Cell to cell adhesion signaling	12.50%	0.0334	BioCarta
Syndecan-1-mediated signaling events	9.30%	3.16 × 10^−5^	PID
Viral carcinogenesis	7.40%	3.86 × 10^−15^	KEGG
Integrins in angiogenesis	6.30%	0.000143	PID
Beta1 integrin cell surface interactions	6.10%	0.000171	PID
Protein digestion and absorption	4.90%	7.27 × 10^−5^	KEGG
Aurora B signaling	4.90%	0.013	PID
ECM-receptor interaction	4.50%	0.000518	KEGG
Focal adhesion	3.50%	2.10 × 10^−5^	KEGG
Caspase cascade in apoptosis	3.50%	0.0242	PID
Viral myocarditis	3.30%	0.0267	KEGG
AP-1 transcription factor network	2.90%	0.0354	PID
Prolactin	2.90%	0.0354	NetPath
Adherens junction	2.80%	0.0364	KEGG
Alpha6Beta4Integrin	2.80%	0.0364	NetPath
Bacterial invasion of epithelial cells	2.60%	0.0421	KEGG
Proteoglycans in cancer	2.40%	0.00172	KEGG
Platelet activation	2.40%	0.0156	KEGG
Hippo signaling pathway	1.90%	0.0288	KEGG
PI3K-Akt signaling pathway	1.40%	0.0168	KEGG
EGFR1	1.10%	0.043	NetPath

**Table 3:** Pathways involved in upregulated proteins in SCC vs. SIL group using ConsensusPathDB software.

Pathway	Percentage of Proteins (%)	*p*-Value	Pathway Source
Signaling events mediated by HDAC Class III	38.50%	4.05 × 10^−25^	PID
Neutrophil extracellular trap formation	28.90%	3.72 × 10^−90^	KEGG
Hemoglobins chaperone	23.10%	4.04 × 10^−5^	BioCarta
Viral carcinogenesis	16.20%	1.31 × 10^−41^	KEGG
Necroptosis	13.20%	3.96 × 10^−24^	KEGG
Cell to cell adhesion signaling	12.50%	0.042	BioCarta
Viral myocarditis	3.30%	0.0409	KEGG

**Table 4:** Pathways involved in upregulated and downregulated proteins in SCC vs. normal cervix group using ConsensusPathDB software.

Upregulated Proteins			
Pathway	Percentage of Proteins (%)	*p*-Value	Pathway Source
Neutrophil extracellular trap formation	28.9%	9.73 × 10^−102^	KEGG
Viral carcinogenesis	15.7%	7.71 × 10^−44^	KEGG
Signaling events mediated by HDAC Class III	13.2%	1.97 × 10^−26^	KEGG
Necroptosis	4.0%	0.0196	KEGG
Viral myocarditis	2.8%	0.0375	KEGG
Adherens junction	2.6%	0.0425	KEGG
Bacterial invasion of epithelial cells	2.6%	0.0435	KEGG
**Downregulated proteins**			
VEGFR3 signaling in lymphatic endothelium	8.00%	9.93 × 10^−6^	PID
Syndecan-1-mediated signaling events	7.00%	3.04 × 10^−8^	PID
Integrins in angiogenesis	4.80%	9.78 × 10^−8^	PID
Beta1 integrin cell surface interactions	4.50%	1.13 × 10^−7^	PID
Beta3 integrin cell surface interactions	4.50%	3.13 × 10^−5^	PID
ECM-receptor interaction	3.40%	2.70 × 10^−7^	KEGG
IL4-mediated signaling events	3.10%	6.87 × 10^−5^	PID
Protein digestion and absorption	2.90%	4.36 × 10^−7^	KEGG
Platelet activation	1.60%	0.00025	KEGG
Relaxin signaling pathway	1.60%	0.00027	KEGG
Focal adhesion	1.50%	3.28 × 10^−6^	KEGG
Proteoglycans in cancer	1.00%	0.00069	KEGG
Human papillomavirus infection	0.90%	1.47 × 10^−5^	KEGG
PI3K-Akt signaling pathway	0.80%	1.81 × 10^−5^	KEGG

**Table 5:** Comparison of ANXA2 and CRNN histoscores between the histological groups.

ANXA2 Histoscore						CRNN Histoscore					
Parameters	Negative (n)	Low (n)	High (n)	Total (n)	*p*-Value	Parameters	Negative (n)	Low (n)	High (n)	Total (n)	*p*-Value
Age (years)						Age (years)					
<50	3	4	64	71	0.043	<50	14	28	30	72	0.552
≥50	9	1	44	54		≥50	7	25	22	54	
											
SIL grade						SIL grade					
1	0	0	10	10	0.110	1	1	1	8	10	0.010
2	0	0	4	4		2	1	0	3	4	
3	0	2	5	7		3	2	5	0	7	
											
SCC stage						SCC stage					
Stage I	1	2	27	30	0.444	Stage I	6	13	11	30	0.095
Stage IA	0	0	1	1		Stage IA	0	0	1	1	
Stage IB	3	0	19	22		Stage IB	4	9	9	22	
Stage IC	0	0	1	1		Stage IC	1	0	0	1	
Stage II	0	0	6	6		Stage II	0	5	1	6	
Stage IIA	0	0	19	19		Stage IIA	0	13	6	19	
Stage IIB	0	0	11	11		Stage IIB	3	6	3	12	
Stage III	0	0	1	1		Stage III	1	0	0	1	
Stage IIIB	0	1	3	4		Stage IIIB	2	1	1	4	

Chi-square test, statistical significance set at *p*-value ≤ 0.05.

**Table 6:** The association between ANXA2 and CRNN proteins expression with subject’s age, SIL grade, and SCC stage.

Histological Group	Negative n	Histoscore Low n	High n	Total n	Comparison between all Histological Groups *p*-Value	Histological Group vs. Normal Cervix *p*-Value	SCC vs. SIL *p*-Value
**ANXA2 protein**							
Normal cervix	1	0	7	8			
SIL	0	2	19	21	0.351	0.183	
SCC	4	3	89	96		0.512	0.281
Total	5	5	115	125			
**CRNN protein**							
Normal cervix	0	0	8	8			
SIL	4	6	11	21	0.004	0.055	
SCC	18	47	33	98		0.001	0.212
Total	22	53	52	127			

Chi-square test, statistical significance set at *p*-value ≤ 0.05.

## Data Availability

The data presented in this study are available on request from the corresponding author.

## Supplementary Materials

The following supporting information can be downloaded at: https://www.mdpi.com/article/10.3390/cimb45040235/s1, Figure S1: Differentially expressed proteins between the normal cervix, HPV-associated SIL, and HPV-associated SCC groups. Figure S2: Classification of molecular functions of downregulated proteins in SIL vs. normal cervix group. The percentage reflects the number of proteins involved. The gene ontology classifications were generated using DAVID version 6.8 (released 19 February 2022). Figure S3: Classification of protein–protein interactions of downregulated proteins in SIL vs. normal cervix group. Protein–protein interaction networks were performed using STRING analysis. The colored and filled nodes are query proteins with predicted 3D structure. The predicted interactions are represented by the colored lines; green—gene neighborhood; red—gene fusion; blue—gene co-occurrence; yellow—text mining; black—co-expression; and purple—protein homology. Figure S4: Classification of molecular functions of upregulated proteins in the SCC vs. SIL group. The percentage reflects the number of proteins involved. The gene ontology classifications were generated using DAVID version 6.8 (released 19 February 2022). Figure S5: Classification of protein–protein interactions of upregulated proteins in the SCC vs. SIL group. Protein–protein interaction networks were performed using STRING analysis. The colored and filled nodes are query proteins with predicted 3D structure. The predicted interactions are represented by the colored lines; green—gene neighborhood; red—gene fusion; blue—gene co-occurrence; yellow—text mining; black—co-expression; and purple—protein homology. Figure S6: Classification of molecular functions of upregulated proteins in SCC vs. normal cervix group. The percentage reflects the number of proteins involved in each molecular function. The gene ontology classifications were generated using DAVID version 6.8 (released 19 February 2022). Figure S7: Classification of protein–protein interactions of upregulated proteins in SCC vs. normal group. Protein–protein interaction networks were performed using STRING analysis. The colored and filled nodes are query proteins with predicted 3D structure. The predicted interactions are represented by the colored lines; green—gene neighborhood; red—gene fusion; blue—gene co-occurrence; yellow—text mining; black—co-expression; and purple—protein homology. Figure S8: Classification of molecular functions of downregulated proteins in SCC vs. normal cervix group. The percentage reflects the number of proteins involved in each molecular function. The gene ontology classifications were generated using DAVID version 6.8 (released 19 February 2022). Figure S9: Classification of protein–protein interactions of downregulated proteins in SCC vs. normal group. Protein–protein interaction networks were performed using STRING analysis. The colored and filled nodes are query proteins with predicted 3D structure. The predicted interactions are represented by the colored lines; green—gene neighborhood; red—gene fusion; blue—gene co-occurrence; yellow—text mining; black—co-expression; and purple—protein homology. Table S1: Semi-quantitative scoring method for immunohistochemical expression of ANXA2 and CRNN proteins. Table S2: Combined list of proteins identified from orbitrap and timsTOF MS. Table S3: Lists of the differentially expressed proteins between the normal cervix, HPV-associated SIL and HPV-associated SCC groups. Table S4: Demographic and pathology information of the samples obtained from the Department of Pathology, School of Medical Sciences, Universiti Sains Malaysia (USM) Kubang Kerian, Kelantan, Malaysia. Table S5: Tissue microarray specification of BB10011 and CR1101, Biomax Inc., USA.
