# Supplementary material for: Protein Profiling in Human Papillomavirus-Associated Cervical Carcinogenesis: Cornulin as a Biomarker for Disease Progression
Source: Curr Issues Mol Biol. 2023 Apr 20;45(4):3603–27. doi: 10.3390/cimb45040235 (PMC10137006; doi:10.3390/cimb45040235)
Supplement: Supplementary file 1 [file cimb-45-00235-s001.zip › Supplementary Figures.pdf]

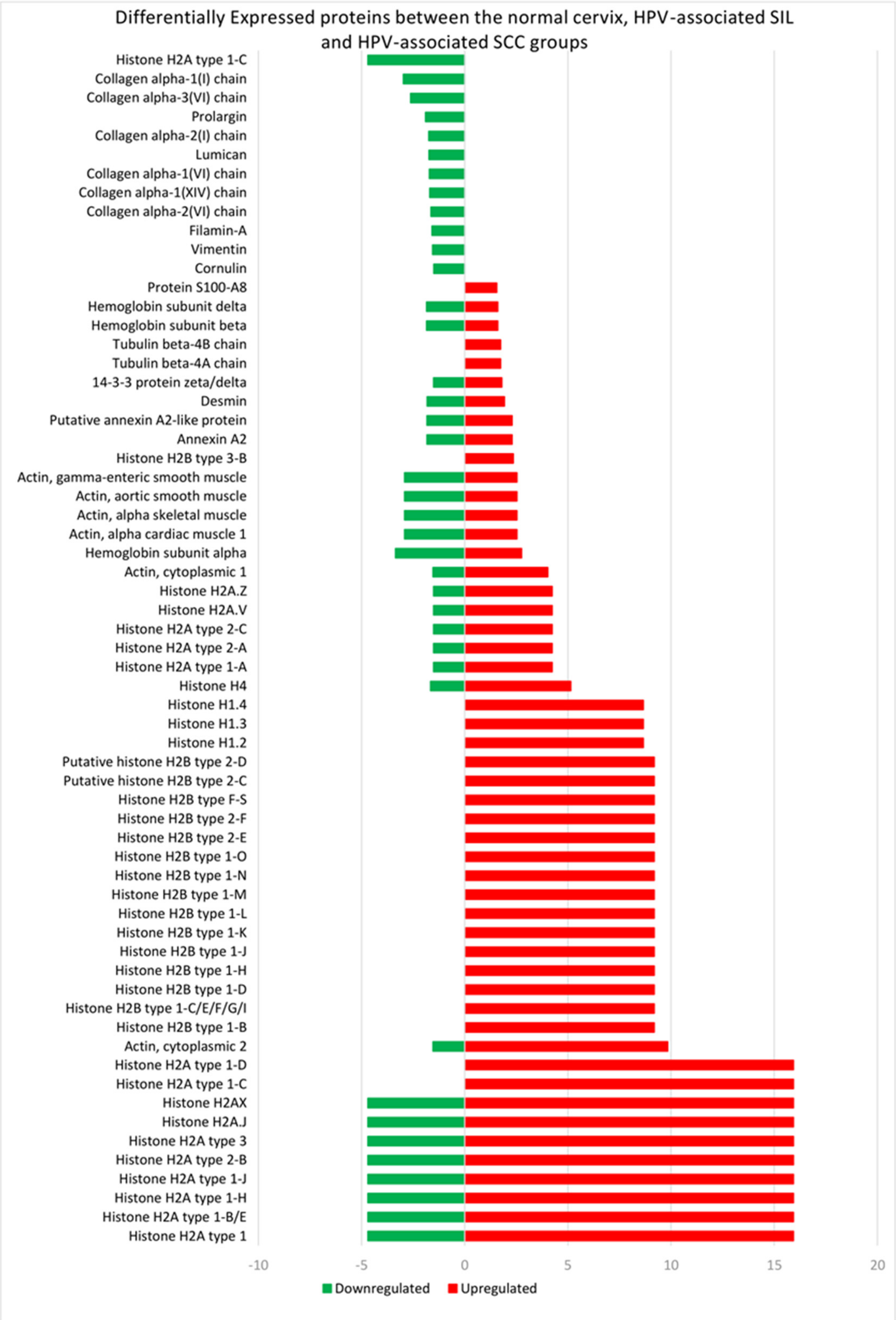

Figure S1 Differentially expressed proteins between the normal cervix, HPV-associated SIL and HPV-associated SCC groups.

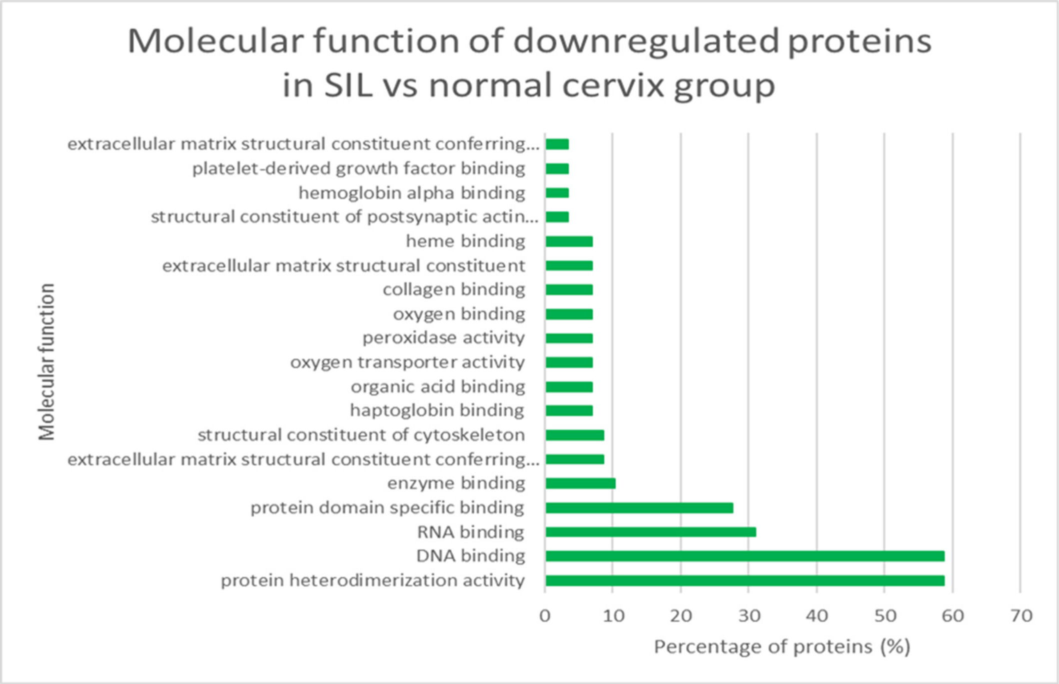

Figure S2 Classification of molecular functions of downregulated proteins in SIL vs Normal cervix group. The percentage reflects the number of proteins involved. The gene ontology classifications were generated using DAVID version 6.8 (released 2022-02-19).

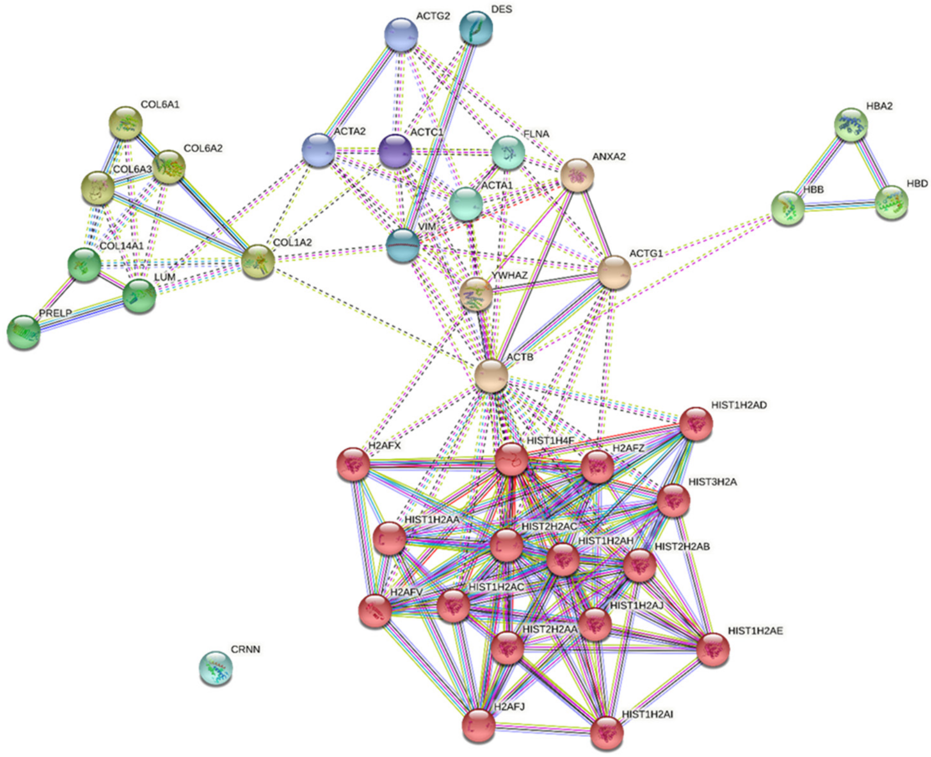

Figure S3 Classification of protein-protein interactions of downregulated proteins in SIL vs Normal cervix group. Protein-protein interaction networks were done using STRING analysis. The colored and filled nodes are query proteins with predicted 3D structure. The predicted interactions are represented by the colored lines; green – gene neighborhood; red- gene fusion; blue- gene co-occurrence; yellow- text mining; black- co-expression and purple- protein homology.

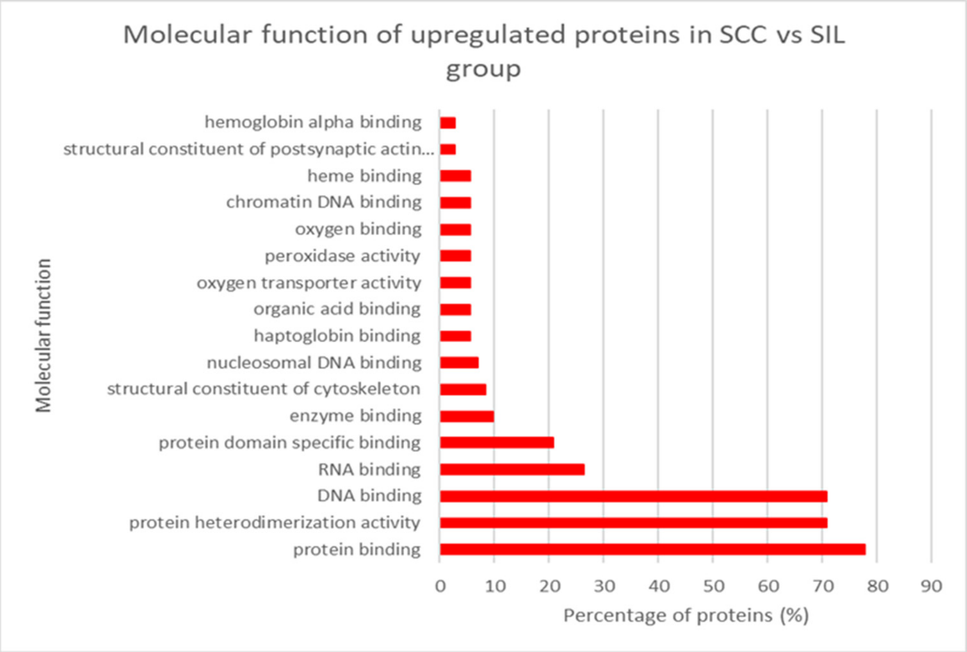

Figure S4 Classification of molecular functions of upregulated proteins in the SCC vs SIL group. The percentage reflects the number of proteins involved. The gene ontology classifications were generated using DAVID version 6.8 (released 2022-02-19).

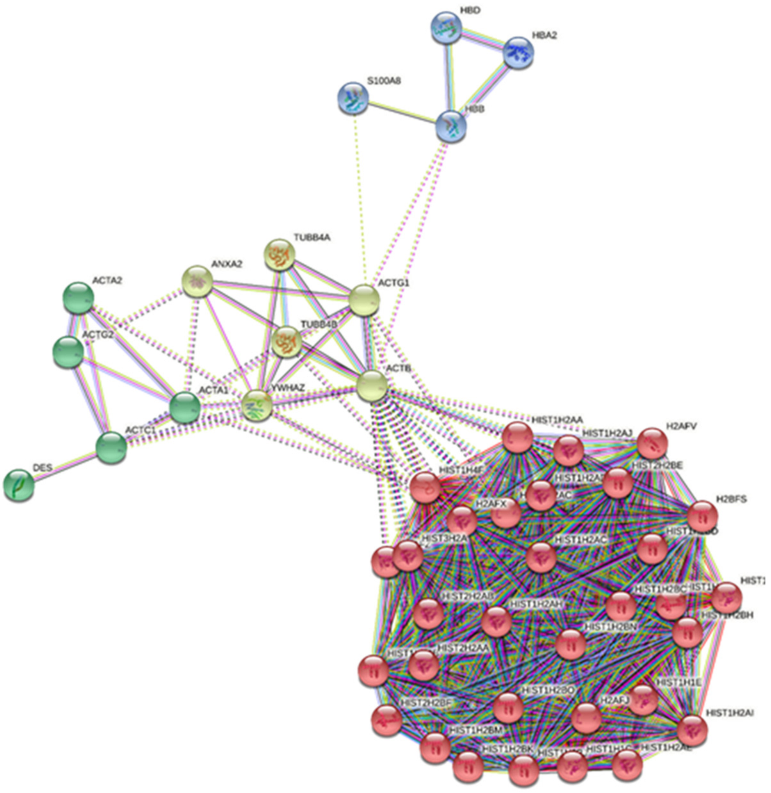

Figure S5 Classification of protein-protein interactions of upregulated proteins in the SCC vs SIL group. Protein-protein interaction networks were done using STRING analysis. The colored and filled nodes are query proteins with predicted 3D structure. The predicted interactions are represented by the colored lines; green – gene neighborhood; red- gene fusion; blue- gene co-occurrence; yellow-text mining; black- co-expression and purple- protein homology.

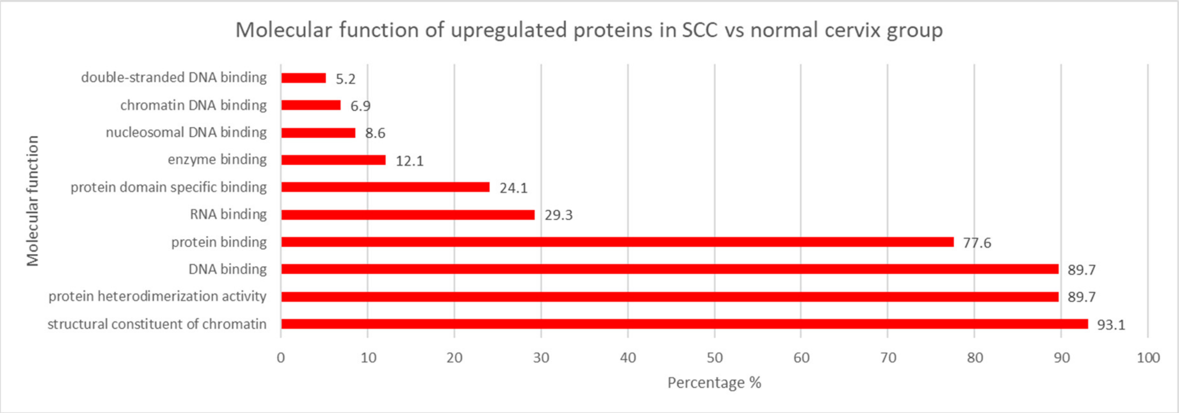

Figure S6 Classification of molecular functions of upregulated proteins in SCC vs normal cervix group. The percentage reflects the number of proteins involved in each molecular function. The gene ontology classifications were generated using DAVID version 6.8 (released 2022-02-19).

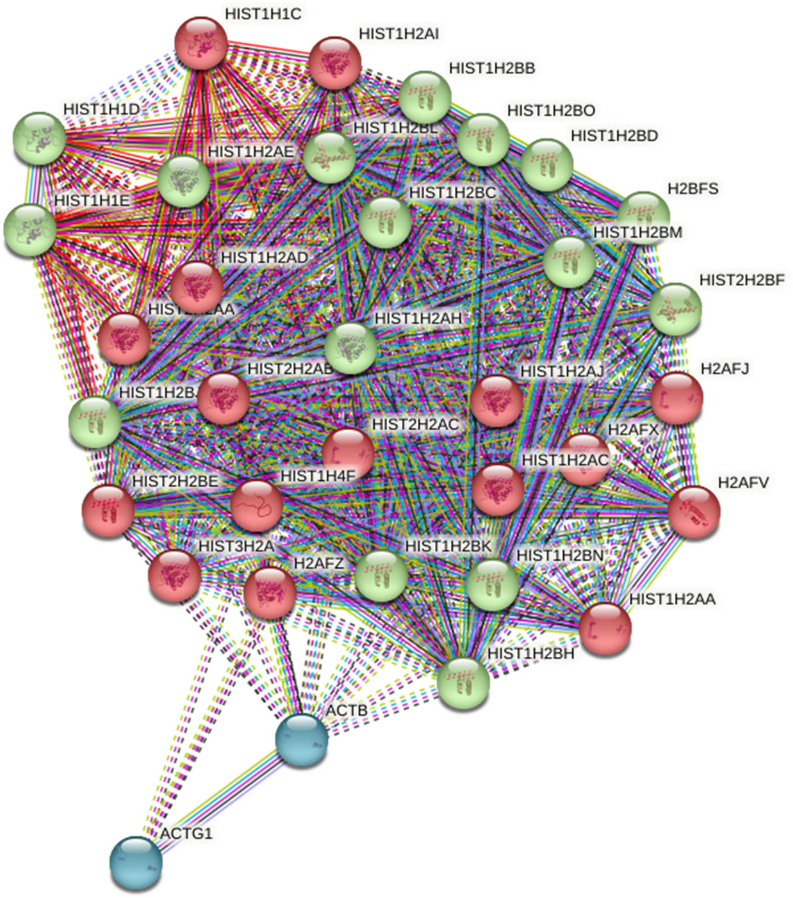

Figure S7 Classification of protein-protein interactions of upregulated proteins in SCC vs normal group. Protein-protein interaction networks were done using STRING analysis. The colored and filled nodes are query proteins with predicted 3D structure. The predicted interactions are represented by the colored lines; green – gene neighborhood; red- gene fusion; blue- gene co-occurrence; yellow- text mining; black- co-expression and purple- protein homology.

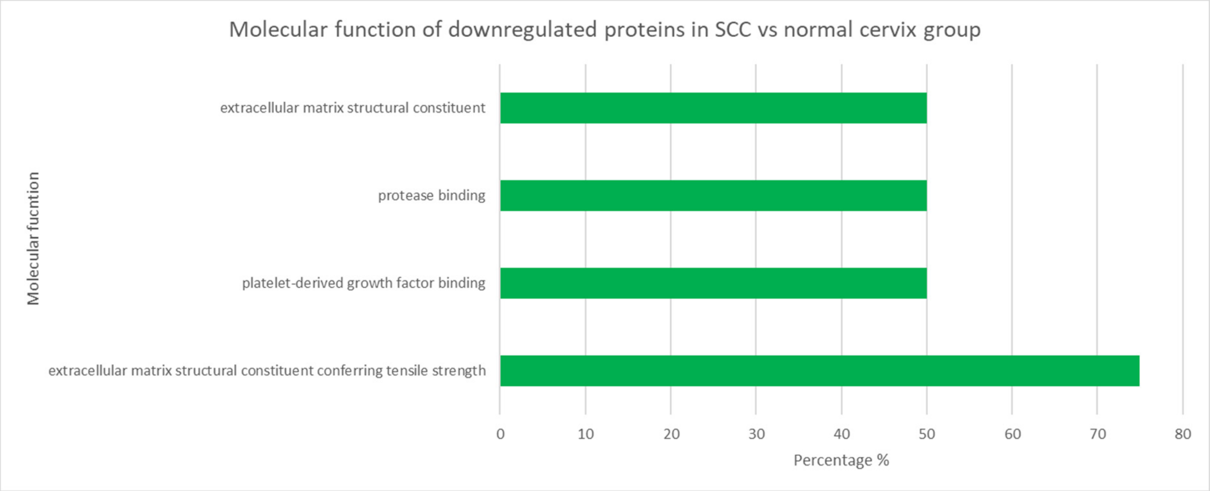

Figure S8 Classification of molecular functions of downregulated proteins in SCC vs normal cervix group. The percentage reflects the number of proteins involved in each molecular function. The gene ontology classifications were generated using DAVID version 6.8 (released 2022-02-19).

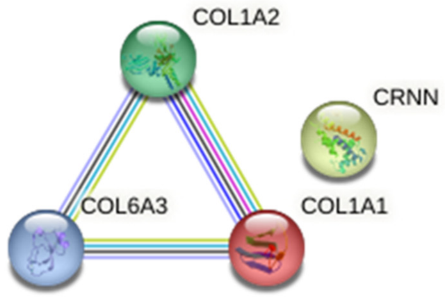

Figure S9 Classification of protein-protein interactions of downregulated proteins in SCC vs normal group. Protein-protein interaction networks were done using STRING analysis. The colored and filled nodes are query proteins with predicted 3D structure. The predicted interactions are represented by the colored lines; green – gene neighborhood; red- gene fusion; blue- gene co-occurrence; yellow- text mining; black- co-expression and purple- protein homology.
