# Supplementary material for: Protein Profiling in Human Papillomavirus-Associated Cervical Carcinogenesis: Cornulin as a Biomarker for Disease Progression
Source: Curr Issues Mol Biol. 2023 Apr 20;45(4):3603–27. doi: 10.3390/cimb45040235 (PMC10137006; doi:10.3390/cimb45040235)
Supplement: Supplementary file 1 [file cimb-45-00235-s001.zip › Supplementary Tables.pdf]

Table S1 Semi-quantitative scoring method for immunohistochemical expression of ANXA2 and CRNN proteins

Percentage of positive cells	Score	Staining intensity	Score
< 1% positive cells	0	No staining	0
1–25% positive cells	1	Weak staining	1
26–50% positive cells	2	Moderate staining	2
51–75% positive cells	3	Strong staining	3
≥ 75% positive cells	4		

Final score = percentage of positive cells score x staining intensity score.

Final histoscore: 0 = negative.

1 to 3/12 = low expression.

≥ 4/12 = high expression.

Table S3a List of differentially expressed proteins in squamous intraepithelial lesion (SIL) versus normal cervix

Protein names	Mol. weight [kDa]	Fold change
Histone H2A type 3<br>Histone H2A type 1-C<br>Histone H2A type 1<br>Histone H2A type 1-H<br>Histone H2A type 1-B/E<br>Histone H2A type 1-J<br>Histone H2A type 2-B<br>Histone H2A.J<br>Histone H2AX	14.135	-4.70137
Hemoglobin subunit alpha	15.257	-3.3627
Collagen alpha-1(I) chain	138.03	-2.98011
Actin, aortic smooth muscle<br>Actin, gamma-enteric smooth muscle<br>Actin, alpha cardiac muscle 1<br>Actin, alpha skeletal muscle	42.009	-2.91875
Collagen alpha-3(VI) chain	343.67	-2.62217
Prolargin	43.809	-1.89943
Hemoglobin subunit beta<br>Hemoglobin subunit delta	15.998	-1.85774
Annexin A2<br>Putative annexin A2-like protein	38.604	-1.83448
Desmin	53.535	-1.82973
Collagen alpha-2(I) chain	129.31	-1.75035
Lumican	38.429	-1.74055
Collagen alpha-1(VI) chain	108.53	-1.71452
Collagen alpha-1(XIV) chain	193.51	-1.69579
Histone H4	11.367	-1.66344
Collagen alpha-2(VI) chain	108.58	-1.63643
Filamin-A	280.74	-1.59287
Vimentin	53.651	-1.55866
Actin, cytoplasmic 2<br>Actin, cytoplasmic 1	41.792	-1.54039
Histone H2A type 2-C<br>Histone H2A type 2-A<br>Histone H2A type 1-A<br>Histone H2A.V<br>Histone H2A.Z	14.107	-1.51746
14-3-3 protein zeta/delta	27.745	-1.51517
Cornulin	53.533	-1.50442

Table S3b List of differentially expressed proteins in squamous cell carcinoma (SCC) versus squamous intraepithelial lesion (SIL)

Protein names	Mol. weight [kDa]	Fold change
Histone H2A type 3<br>Histone H2A type 1-C<br>Histone H2A type 1<br>Histone H2A type 1-D<br>Histone H2A type 1-H<br>Histone H2A type 1-B/E<br>Histone H2A type 1-J<br>Histone H2A type 2-B<br>Histone H2A.J<br>Histone H2AX	14.135	15.94075
Actin, cytoplasmic 2	41.792	9.840621
Histone H2B type 2-E<br>Histone H2B type 1-M<br>Histone H2B type 1-N<br>Histone H2B type 1-H<br>Histone H2B type 2-F<br>Histone H2B type 1-C/E/F/G/I<br>Histone H2B type 1-D<br>Histone H2B type F-S<br>Histone H2B type 1-B<br>Histone H2B type 1-O<br>Histone H2B type 1-J<br>Histone H2B type 1-K<br>Putative histone H2B type 2-D<br>Histone H2B type 1-L<br>Putative histone H2B type 2-C	13.948	9.180867
Histone H1.3<br>Histone H1.2<br>Histone H1.4	22.35	8.654992
Histone H4	11.367	5.144723
Histone H2A type 2-C<br>Histone H2A type 2-A<br>Histone H2A type 1-A<br>Histone H2A.V<br>Histone H2A.Z	14.107	4.241194
Actin, cytoplasmic 1	41.792	4.024891
Hemoglobin subunit alpha	15.257	2.757901
Actin, aortic smooth muscle<br>Actin, gamma-enteric smooth muscle<br>Actin, alpha cardiac muscle 1<br>Actin, alpha skeletal muscle	42.009	2.535343
Histone H2B type 3-B	13.95	2.359147
Annexin A2<br>Putative annexin A2-like protein	38.604	2.3024

Desmin	53.535	1.927182
14-3-3 protein zeta/delta	27.745	1.798218
Tubulin beta-4B chain<br>Tubulin beta-4A chain	49.83	1.745452
Hemoglobin subunit beta<br>Hemoglobin subunit delta	15.998	1.606587
Protein S100-A8	10.834	1.552563

Table S3c List of upregulated and downregulated proteins in squamous cell carcinoma (SCC) versus normal cervix.

**Upregulated proteins**		
**Protein names**	**Mol. weight [kDa]**	**Fold change**
Actin, cytoplasmic 2<br>Actin, cytoplasmic 2, N-terminally processed	41.792	13.28935
Histone H2A type 3<br>Histone H2A type 1-C<br>Histone H2A type 1<br>Histone H2A type 1-D<br>Histone H2A type 1-H<br>Histone H2A type 1-B/E<br>Histone H2A type 1-J<br>Histone H2A type 1-F<br>Histone H2A type 1-K<br>Histone H2A type 2-B<br>Histone H2A.J<br>Histone H2AX	14.135	11.23938
Histone H2B 3<br>Histone H2B type 2-E<br>Histone H2B type 1-M<br>Histone H2B type 1-N<br>Histone H2B type 1-H<br>Histone H2B type 1-C/E/G<br>Histone H2B type 2-B<br>Histone H2B type 2-F<br>Histone H2B type 1-C/E/F/G/I<br>Histone H2B type 1-D<br>Histone H2B type F-S<br>Histone H2B type 1-B<br>Histone H2B type 1-O<br>Histone H2B type 1-J<br>Histone H2B type 1-K<br>Putative histone H2B type 2-D<br>Histone H2B type 1-A<br>Histone H2B type 1-L<br>Histone H2B type 1-F/J/L<br>Histone H2B type 1-P<br>Putative histone H2B type 2-C	13.948	9.180867
Histone H1.3	22.35	8.654992

Histone H1.2<br>Histone H1.4		
Histone H4	11.367	3.481285
Histone H2A type 2-C<br>Histone H2A type 2-A<br>Histone H2A type 1-A<br>Histone H2A-alpha<br>Histone H2A.V<br>Histone H2A.Z	14.107	2.723737
Actin, cytoplasmic 1<br>Actin, cytoplasmic 1, N-terminally processed	41.792	2.484502
Histone H2B type 3-A<br>Histone H2B type 3-B	13.95	1.726984
**Downregulated proteins**		
Collagen alpha-3(VI) chain	343.67	-2.96006
Collagen alpha-2(I) chain	129.31	-2.67062
Collagen alpha-1(I) chain	138.03	-2.65007
Cornulin	53.533	-2.53514

Table S4 Demographic and pathology information of the samples obtained from the Department of Pathology, School of Medical Sciences, Universiti Sains Malaysia (USM) Kubang Kerian, Kelantan, Malaysia.

No	Lab number	Histopathological Diagnosis	Patient age (years)	SIL grade	SCC stage
**1**	P793/16(1)	Normal cervix	54	-	-
**2**	P2004/16(A2)	Normal cervix	52	-	-
**3**	P3825/18(2)	Normal cervix	56	-	-
**4**	P2127/19(2)	Normal cervix	57	-	-
**5**	P4475/19(1)	Normal cervix	41	-	-
**6**	P4380/19(A1)	Normal cervix	57	-	-
**7**	P733/19(5)	SIL	28	Grade I	-
**8**	P611/16(C)	SIL	36	Grade III	-
**9**	P619/15(3)	SIL	49	Grade III	-
**10**	P1952/15(3)	SIL	41	Grade III	-
**11**	P1386/13(A1)	SIL	48	Grade III	-
**12**	P2075/16	SCC	51	-	Stage IIB
**13**	P3108/16(1)	SCC	91	-	Stage IIIA
**14**	P2233/20	SCC	48	-	Stage IB1
**15**	P2646/13(B)	SCC	63	-	Stage IB1
**16**	P195/14(A2)	SCC	70	-	Stage IIB
**17**	P3698/14(B1)	SCC	55	-	Stage IIA

Table S5 Tissue microarray specification of BB10011 and CR1101, Biomax Inc, USA

Specification of tissue microarray BB10011

Position	No.	Age	Sex	Organ/Anatomic Site	Pathology diagnosis	TNM	Grade	Stage	Type	Tissue ID.
**A1**	1	40	F	Cervix	Cervical intraepithelial neoplasia	-	1	-	CIN	Fdu021939
**A2**	2	40	F	Cervix	Cervical intraepithelial neoplasia	-	1	-	CIN	Fdu021939
**A3**	3	47	F	Cervix	Cervical intraepithelial neoplasia	-	1	-	CIN	Fdu020509
**A4**	4	47	F	Cervix	Cervical intraepithelial neoplasia	-	1	-	CIN	Fdu020509
**A5**	5	43	F	Cervix	Cervical intraepithelial neoplasia	-	1	-	CIN	Fdu060215
**A6**	6	43	F	Cervix	Cervical intraepithelial neoplasia	-	1	-	CIN	Fdu060215
**A7**	7	58	F	Cervix	Cervical intraepithelial neoplasia	-	-	-	CIN	Fdu050684
**A8**	8	58	F	Cervix	Cervical intraepithelial neoplasia	-	1	-	CIN	Fdu050684
**B1**	9	38	F	Cervix	Cervical intraepithelial neoplasia	-	1	-	CIN	Fdu050583
**B2**	10	38	F	Cervix	Cervical intraepithelial neoplasia	-	1	-	CIN	Fdu050583
**B3**	11	53	F	Cervix	Cervical intraepithelial neoplasia	-	-	-	CIN	Fdu021993
**B4**	12	53	F	Cervix	Cervical intraepithelial neoplasia with epidermal erosion	-	1	-	CIN	Fdu021993
**B5**	13	44	F	Cervix	Cervical intraepithelial neoplasia	-	1	-	CIN	Fdu060278
**B6**	14	44	F	Cervix	Cervical intraepithelial neoplasia	-	1	-	CIN	Fdu060278
**B7**	15	44	F	Cervix	Cervical intraepithelial neoplasia	-	1	-	CIN	Fdu050612
**B8**	16	44	F	Cervix	Cervical intraepithelial neoplasia	-	1	-	CIN	Fdu050612
**C1**	17	40	F	Cervix	Cervical intraepithelial neoplasia	-	3	-	CIN	Fdu060279
**C2**	18	40	F	Cervix	Cervical intraepithelial neoplasia	-	3	-	CIN	Fdu060279
**C3**	19	32	F	Cervix	Cervical intraepithelial neoplasia	-	2	-	CIN	Fdu021238
**C4**	20	32	F	Cervix	Cervical intraepithelial neoplasia	-	2	-	CIN	Fdu021238
**C5**	21	66	F	Cervix	Cervical intraepithelial neoplasia	-	1	-	CIN	Fur010400
**C6**	22	66	F	Cervix	Cervical intraepithelial neoplasia	-	1	-	CIN	Fur010400
**C7**	23	51	F	Cervix	Cervical intraepithelial neoplasia	-	-	-	CIN	Fur060453

**C8**	24	51	F	Cervix	Cervical intraepithelial neoplasia with epidermal erosion	-	3	-	CIN	Fur060453
**D1**	25	42	F	Cervix	Cervical intraepithelial neoplasia	-	1	-	CIN	Fdu050665
**D2**	26	42	F	Cervix	Cervical intraepithelial neoplasia with epidermal erosion	-	1	-	CIN	Fdu050665
**D3**	27	45	F	Cervix	Cervical intraepithelial neoplasia	-	2	-	CIN	Fdu030335
**D4**	28	45	F	Cervix	Cervical intraepithelial neoplasia	-	2	-	CIN	Fdu030335
**D5**	29	60	F	Cervix	Cervical intraepithelial neoplasia	-	3	-	CIN	Fdu030441
**D6**	30	60	F	Cervix	Cervical intraepithelial neoplasia	-	3	-	CIN	Fdu030441
**D7**	31	47	F	Cervix	Cervical intraepithelial neoplasia	-	3	-	CIN	Fdu021150
**D8**	32	47	F	Cervix	Cervical intraepithelial neoplasia	-	3	-	CIN	Fdu021150
**E1**	33	47	F	Cervix	Cervical intraepithelial neoplasia	-	3	-	CIN	Fdu050656
**E2**	34	47	F	Cervix	Cervical intraepithelial neoplasia	-	3	-	CIN	Fdu050656
**E3**	35	42	F	Cervix	Cervical intraepithelial neoplasia	-	3	-	CIN	Fdu040991
**E4**	36	42	F	Cervix	Cervical intraepithelial neoplasia	-	3	-	CIN	Fdu040991
**E5**	37	43	F	Cervix	Cervical intraepithelial neoplasia	-	2	-	CIN	Fur050759
**E6**	38	43	F	Cervix	Cervical intraepithelial neoplasia	-	2	-	CIN	Fur050759
**E7**	39	41	F	Cervix	Cervical intraepithelial neoplasia	-	2	-	CIN	Fdu060182
**E8**	40	41	F	Cervix	Cervical intraepithelial neoplasia	-	2	-	CIN	Fdu060182
**F1**	41	37	F	Cervix	Cervical intraepithelial neoplasia	-	3	-	CIN	Fdu060282
**F2**	42	37	F	Cervix	Cervical intraepithelial neoplasia	-	3	-	CIN	Fdu060282
**F3**	43	54	F	Cervix	Squamous cell carcinoma	T1N0M0	1	I	Malignant	Fdu020681
**F4**	44	54	F	Cervix	Squamous cell carcinoma	T1N0M0	1	I	Malignant	Fdu020681
**F5**	45	76	F	Cervix	Squamous cell carcinoma	T2N0M0	2	II	Malignant	Fdu020363
**F6**	46	76	F	Cervix	Squamous cell carcinoma	T2N0M0	2	II	Malignant	Fdu020363
**F7**	47	48	F	Cervix	Squamous cell carcinoma	T1BN0M0	3	IB	Malignant	Fdu020612
**F8**	48	48	F	Cervix	Squamous cell carcinoma	T1BN0M0	3	IB	Malignant	Fdu020612

Specification of tissue microarray CR1101

Position	No.	Age	Sex	Organ/Anatomic Site	Pathology diagnosis	TNM	Grade	Stage	Type	Tissue ID.
**A1**	1	45	F	Cervix	Squamous cell carcinoma	T2AN0M0	1	IIA	Malignant	Fdu050255
**A2**	2	50	F	Cervix	Squamous cell carcinoma	T1N0M0	2	I	Malignant	Fdu050304
**A3**	3	48	F	Cervix	Squamous cell carcinoma	T1N0M0	2	I	Malignant	Fdu050087
**A4**	4	55	F	Cervix	Squamous cell carcinoma	T3N1M0	2	IIIB	Malignant	Fdu050086
**A5**	5	41	F	Cervix	Squamous cell carcinoma	T1N0M0	1	I	Malignant	Fdu031081
**A6**	6	48	F	Cervix	Squamous cell carcinoma	T2BN0M0	2	IIB	Malignant	Fdu041171
**A7**	7	53	F	Cervix	Squamous cell carcinoma	T2N0M0	2	II	Malignant	Fdu050620
**A8**	8	32	F	Cervix	Squamous cell carcinoma	T1BN0M0	-	IB	Malignant	Fdu041026
**A9**	9	54	F	Cervix	Squamous cell carcinoma	T2BN0M0	2	IIB	Malignant	Fdu041131
**A10**	10	80	F	Cervix	Squamous cell carcinoma	T1N0M0	2	I	Malignant	Fdu030670
**B1**	11	63	F	Cervix	Squamous cell carcinoma	T2AN0M0	3	IIA	Malignant	Fdu030882
**B2**	12	50	F	Cervix	Squamous cell carcinoma	T2BN0M0	3	IIB	Malignant	Fdu041325
**B3**	13	39	F	Cervix	Squamous cell carcinoma	T1N0M0	2	I	Malignant	Fdu041045
**B4**	14	78	F	Cervix	Squamous cell carcinoma	T2AN0M0	2	IIA	Malignant	Fdu031029
**B5**	15	51	F	Cervix	Squamous cell carcinoma	T1BN0M0	2	IB	Malignant	Fdu050231
**B6**	16	32	F	Cervix	Squamous cell carcinoma	T2BN0M0	2	IIB	Malignant	Fdu041031
**B7**	17	41	F	Cervix	Squamous cell carcinoma	T1N0M0	2	I	Malignant	Fdu032098
**B8**	18	58	F	Cervix	Squamous cell carcinoma	T2BN0M0	2	IIB	Malignant	Fdu030689
**B9**	19	50	F	Cervix	Squamous cell carcinoma	T1N0M0	-	I	Malignant	Fdu031649
**B10**	20	63	F	Cervix	Squamous cell carcinoma	T1BN0M0	3	IB	Malignant	Fdu031823
**C1**	21	46	F	Cervix	Squamous cell carcinoma	T2AN0M0	3	IIA	Malignant	Fdu050186
**C2**	22	41	F	Cervix	Squamous cell carcinoma	T2BN0M0	3	IIB	Malignant	Fdu041033
**C3**	23	35	F	Cervix	Squamous cell carcinoma (sparse)	-	-	-	Malignant	Fdu050245
**C4**	24	60	F	Cervix	Squamous cell carcinoma	T1N0M0	3	I	Malignant	Fdu030623
**C5**	25	60	F	Cervix	Squamous cell carcinoma	T2AN0M0	2	IIA	Malignant	Fdu030918
**C6**	26	58	F	Cervix	Squamous cell carcinoma	T2BN0M0	3	IIB	Malignant	Fdu050187
**C7**	27	42	F	Cervix	Squamous cell carcinoma	T2BN0M0	3	IIB	Malignant	Fdu050258

**C8**	28	38	F	Cervix	Squamous cell carcinoma	T1BN0M0	3	IB	Malignant	Fdu031959
**C9**	29	62	F	Cervix	Squamous cell carcinoma	T1CN0M0	3	IC	Malignant	Fdu030657
**C10**	30	45	F	Cervix	Squamous cell carcinoma	T2N0M0	3	II	Malignant	Fdu050328
**D1**	31	42	F	Cervix	Squamous cell carcinoma	T2BN0M0	2	IIB	Malignant	Fdu040492
**D2**	32	58	F	Cervix	Squamous cell carcinoma	T2N0M0	2	II	Malignant	Fdu040874
**D3**	33	48	F	Cervix	Squamous cell carcinoma	T2AN0M0	2	IIA	Malignant	Fdu040093
**D4**	34	59	F	Cervix	Squamous cell carcinoma	T2AN0M0	1	IIA	Malignant	Fdu032230
**D5**	35	33	F	Cervix	Squamous cell carcinoma	T1N1M0	1	IIIB	Malignant	Fdu050393
**D6**	36	67	F	Cervix	Squamous cell carcinoma	T1BN0M0	1	IB	Malignant	Fdu032429
**D7**	37	42	F	Cervix	Squamous cell carcinoma	T1BN0M0	-	IB	Malignant	Fdu040875
**D8**	38	40	F	Cervix	Squamous cell carcinoma	T1BN0M0	2	IB	Malignant	Fdu040128
**D9**	39	37	F	Cervix	Squamous cell carcinoma	T2AN0M0	1	IIA	Malignant	Fdu050343
**D10**	40	58	F	Cervix	Squamous cell carcinoma	T2AN0M0	2	IIA	Malignant	Fdu030694
**E1**	41	40	F	Cervix	Squamous cell carcinoma	T1N0M0	2	I	Malignant	Fdu030467
**E2**	42	46	F	Cervix	Squamous cell carcinoma	T1BN1M0	2	IIIB	Malignant	Fdu031222
**E3**	43	67	F	Cervix	Squamous cell carcinoma	T1BN0M0	-	IB	Malignant	Fdu031576
**E4**	44	36	F	Cervix	Squamous cell carcinoma	T2AN0M0	3	IIA	Malignant	Fdu050136
**E5**	45	48	F	Cervix	Squamous cell carcinoma	T2AN0M0	2	IIA	Malignant	Fdu060001
**E6**	46	42	F	Cervix	Squamous cell carcinoma	T1N0M0	3	I	Malignant	Fdu050080
**E7**	47	42	F	Cervix	Squamous cell carcinoma	T2BN0M0	3	IIB	Malignant	Fdu041266
**E8**	48	41	F	Cervix	Squamous cell carcinoma	T2AN0M0	3	IIA	Malignant	Fdu041301
**E9**	49	55	F	Cervix	Squamous cell carcinoma	T1N0M0	3	I	Malignant	Fdu031543
**E10**	50	61	F	Cervix	Squamous cell carcinoma	T1BN0M0	3	IB	Malignant	Fdu041326
**F1**	51	48	F	Cervix	Squamous cell carcinoma	T1N0M0	3	I	Malignant	Fdu032401
**F2**	52	39	F	Cervix	Squamous cell carcinoma	T2N0M0	3	II	Malignant	Fdu050590
**F3**	53	46	F	Cervix	Squamous cell carcinoma (sparse)	T1N0M0	3	I	Malignant	Fdu031555
**F4**	54	71	F	Cervix	Squamous cell carcinoma	T1BN0M0	3	IB	Malignant	Fdu031573
**F5**	55	46	F	Cervix	Squamous cell carcinoma	T2BN0M0	3	IIB	Malignant	Fdu032031
**F6**	56	67	F	Cervix	Squamous cell carcinoma	T1N0M0	3	I	Malignant	Fdu050313
**F7**	57	50	F	Cervix	Squamous cell carcinoma	T1BN0M0	3	IB	Malignant	Fdu050135
**F8**	58	40	F	Cervix	Squamous cell carcinoma	T2AN0M0	3	IIA	Malignant	Fdu031179

**F9**	59	38	F	Cervix	Squamous cell carcinoma	T1N0M0	3	I	Malignant	Fdu031388
**F10**	60	72	F	Cervix	Squamous cell carcinoma	T2AN0M0	3	IIA	Malignant	Fdu050157
**G1**	61	30	F	Cervix	Squamous cell carcinoma	T1AN0M0	3	IA	Malignant	Fdu050037
**G2**	62	51	F	Cervix	Squamous cell carcinoma	T1BN0M0	3	IB	Malignant	Fdu050830
**G3**	63	40	F	Cervix	Squamous cell carcinoma	T1N0M0	3	I	Malignant	Fdu041331
**G4**	64	50	F	Cervix	Squamous cell carcinoma	T1N0M0	3	I	Malignant	Fdu030548
**G5**	65	39	F	Cervix	Squamous cell carcinoma	T3N0M0	3	III	Malignant	Fdu050252
**G6**	66	45	F	Cervix	Squamous cell carcinoma	T1N1M0	3	IIIB	Malignant	Fdu050023
**G7**	67	39	F	Cervix	Squamous cell carcinoma	T2BN0M0	3	IIB	Malignant	Fdu050041
**G8**	68	50	F	Cervix	Squamous cell carcinoma	T1BN0M0	3	IB	Malignant	Fdu050168
**G9**	69	47	F	Cervix	Squamous cell carcinoma	T1BN0M0	3	IB	Malignant	Fdu031647
**G10**	70	44	F	Cervix	Squamous cell carcinoma	T1N0M0	3	I	Malignant	Fdu032033
**H1**	71	49	F	Cervix	Squamous cell carcinoma	T1N0M0	3	I	Malignant	Fdu032415
**H2**	72	43	F	Cervix	Squamous cell carcinoma	T1BN0M0	2	IB	Malignant	Fdu040322
**H3**	73	49	F	Cervix	Squamous cell carcinoma	T2AN0M0	3	IIA	Malignant	Fdu032247
**H4**	74	50	F	Cervix	Squamous cell carcinoma	T2AN0M0	3	IIA	Malignant	Fdu050337
**H5**	75	50	F	Cervix	Squamous cell carcinoma	T1N0M0	3	I	Malignant	Fdu030591
**H6**	76	42	F	Cervix	Squamous cell carcinoma	T2AN0M0	3	IIA	Malignant	Fdu041260
**H7**	77	44	F	Cervix	Squamous cell carcinoma	T1BN0M0	3	IB	Malignant	Fdu041259
**H8**	78	57	F	Cervix	Squamous cell carcinoma	T1BN0M0	3	IB	Malignant	Fdu032209
**H9**	79	40	F	Cervix	Squamous cell carcinoma	T1N0M0	3	I	Malignant	Fdu041291
**H10**	80	53	F	Cervix	Squamous cell carcinoma	T1BN0M0	3	IB	Malignant	Fdu031345
**I1**	81	50	F	Cervix	Squamous cell carcinoma	T2N0M0	2	II	Malignant	Fdu040488
**I2**	82	48	F	Cervix	Squamous cell carcinoma	T1BN0M0	2	IB	Malignant	Fdu032482
**I3**	83	42	F	Cervix	Squamous cell carcinoma	T1N0M0	3	I	Malignant	Fdu040138
**I4**	84	40	F	Cervix	Squamous cell carcinoma	T1N0M0	3	I	Malignant	Fdu040302
**I5**	85	38	F	Cervix	Squamous cell carcinoma	T2AN0M0	3	IIA	Malignant	Fdu032381
**I6**	86	44	F	Cervix	Squamous cell carcinoma	T1BN0M0	3	IB	Malignant	Fdu032527
**I7**	87	58	F	Cervix	Squamous cell carcinoma	T1N0M0	3	I	Malignant	Fdu040254
**I8**	88	55	F	Cervix	Squamous cell carcinoma	T1N0M0	3	I	Malignant	Fdu040032
**I9**	89	67	F	Cervix	Squamous cell carcinoma	T1N0M0	3	I	Malignant	Fdu040075
**I10**	90	68	F	Cervix	Squamous cell carcinoma	T2AN0M0	3	IIA	Malignant	Fdu032417

**J1**	91	29	F	Cervix	Squamous cell carcinoma	T1N0M0	3	I	Malignant	Fdu031227
**J2**	92	54	F	Cervix	Squamous cell carcinoma	T1N0M0	3	I	Malignant	Fdu041086
**J3**	93	46	F	Cervix	Squamous cell carcinoma (sparse)	T1BN0M0	-	IB	Malignant	Fdu031194
**J4**	94	55	F	Cervix	Squamous cell carcinoma	T1N0M0	3	I	Malignant	Fdu030692
**J5**	95	45	F	Cervix	Squamous cell carcinoma	T1BN0M0	3	IB	Malignant	Fdu050045
**J6**	96	49	F	Cervix	Adenocarcinoma	T1N0M0	2	I	Malignant	Fdu040462
**J7**	97	46	F	Cervix	Adenocarcinoma	T2BN0M0	2	IIB	Malignant	Fdu041224
**J8**	98	49	F	Cervix	Adenosquamous carcinoma	T1N0M0	-	I	Malignant	Fdu041307
**J9**	99	47	F	Cervix	Endometrioid adenocarcinoma	T1BN0M0	1	IB	Malignant	Fdu032048
**J10**	100	51	F	Cervix	Endometrioid adenocarcinoma	T2AN0M0	1	IIA	Malignant	Fdu040099
**K1**	101	58	F	Cervix	Adjacent chronic cervicitis	-	-	-	NAT	Fdu140266
**K2**	102	61	F	Cervix	Adjacent chronic cervicitis	-	-	-	NAT	Fdu150094
**K3**	103	60	F	Cervix	Adjacent chronic cervicitis	-	-	-	NAT	Fdu140176
**K4**	104	63	F	Cervix	Adjacent chronic cervicitis	-	-	-	NAT	Fdu150376
**K5**	105	70	F	Cervix	Adjacent cervical canals tissue	-	-	-	NAT	Fdu031779
**K6**	106	70	F	Cervix	Adjacent cervical canals tissue	-	-	-	NAT	Fdu031820
**K7**	107	70	F	Cervix	Adjacent cervix tissue	-	-	-	NAT	Fdu031822
**K8**	108	40	F	Cervix	Adjacent cervix tissue	-	-	-	NAT	Fdu031909
**K9**	109	72	F	Cervix	Adjacent cervix tissue	-	-	-	NAT	Fdu031770
**K10**	110	50	F	Cervix	Adjacent cervix tissue	-	-	-	NAT	Fdu032477
